# miR-617 interacts with the promoter of *DDX27* and positively regulates its expression: implications for cancer therapeutics

**DOI:** 10.3389/fonc.2024.1411539

**Published:** 2024-06-13

**Authors:** Neelanjana Sarkar, Radha Mishra, Champaka Gopal, Arun Kumar

**Affiliations:** ^1^ Department of Developmental Biology and Genetics, Indian Institute of Science, Bangalore, India; ^2^ Department of Pathology, Kidwai Memorial Institute of Oncology, Bangalore, India

**Keywords:** microRNA, miRs, miR-617, DDX27, promoter activation, OSCC, cancer, therapeutics

## Abstract

**Background:**

Pervasive transcription of the eukaryotic genome generates noncoding RNAs (ncRNAs), which regulate messenger RNA (mRNA) stability and translation. MicroRNAs (miRNAs/miRs) represent a group of well-studied ncRNAs that maintain cellular homeostasis. Thus, any aberration in miRNA expression can cause diseases, including carcinogenesis. According to microRNA microarray analyses, intronic miR-617 is significantly downregulated in oral squamous cell carcinoma (OSCC) tissues compared to normal oral tissues.

**Methods:**

The miR-617-mediated regulation of *DDX27* is established by performing experiments on OSCC cell lines, patient samples, and xenograft nude mice model. Overexpression plasmid constructs, bisulphite sequencing PCR, bioinformatics analyses, RT-qPCR, Western blotting, dual-luciferase reporter assay, and cell-based assays are utilized to delineate the role of miR-617 in OSCC.

**Results:**

The present study shows that miR-617 has an anti-proliferative role in OSCC cells and is partly downregulated in OSCC cells due to the hypermethylation of its independent promoter. Further, we demonstrate that miR-617 upregulates *DDX27* gene by interacting with its promoter in a dose-dependent and sequence-specific manner, and this interaction is found to be biologically relevant in OSCC patient samples. Subsequently, we show that miR-617 regulates cell proliferation, apoptosis, and anchorage-independent growth of OSCC cells by modulating DDX27 levels. Besides, our study shows that miR-617 exerts its effects through the PI3K/AKT/MTOR pathway via regulating DDX27 levels. Furthermore, the OSCC xenograft study in nude mice shows the anti-tumorigenic potential of miR-617.

**Conclusion:**

miR-617-mediated upregulation of DDX27 is a novel mechanism in OSCC and underscores the therapeutic potential of synthetic miR-617 mimics in cancer therapeutics. To the best of our knowledge, miR-617 is the 15th example of a miRNA that upregulates the expression of a protein-coding gene by interacting with its promoter.

## 1 Introduction

Oral squamous cell carcinoma (OSCC) is the most common malignant epithelial neoplasm of the head and neck region and includes cancer of the lips, labial mucosa, gingiva, buccal mucosa, anterior two-third of the tongue, floor of the mouth, hard palate, and retromolar trigone. It accounts for 84–97% of all oral malignancies (1). In India, it is the second most common cancer having an annual incidence of 1,35,929 cases, preceded by the breast cancer (http://gco.iarc.fr/; 2). It is the most common cancer in males and the fourth most common cancer in females of India (http://gco.iarc.fr/; 2). Despite advances in preventive and treatment strategies, there has been a dismal improvement in the 5-year survival rate for oral cancer in the last few decades, further underscoring the need to identify novel therapeutic targets to increase patient survival and decrease morbidity. Genesis of oral cancer is a sequential process of genetic and epigenetic events, leading to the disruption of normal cellular functions such as cell differentiation, signal transduction, and cell death (3). Among the epigenetic events, downregulation of tumor suppressor miRNAs is one of the causal factors of OSCC (4). Thus, restoration of the levels of tumor suppressor miRNAs can reverse the cancer phenotypes and serve as novel therapeutic targets.

*MIR617*, a primate-specific microRNA gene located at human chromosome 12q21.31, was discovered by Cummins et al. (5) in human colorectal cells. Even after nearly two decades of its discovery, the transcriptional regulation and biological function of this intronic miRNA are largely unexplored. Several miRNA microarray analyses in cancer cell lines and tissues have associated the differential expression of miR-617 with a particular cancer type, but none of these studies has validated its differential expression or explored its role in cancer. For example, miR-617 is one of the 99 miRNAs, which are positively associated with cigarette-smoking rectal cancer patients (6). It is also significantly downregulated in esophageal adenocarcinoma samples compared to their paired normal tissues (7). However, a subsequent study showed that miR-617 is one of the significantly upregulated miRNAs in esophageal cancer cells following treatment with the chemotherapeutic drug 5-fluorouracil (8). Mao et al. (9) showed that miR-617 is significantly downregulated in GH-secreting pituitary adenomas compared to normal pituitary tissues and is associated with chemoresistance in serous ovarian cancer samples (10). In lung cancer, Kim et al. (11) showed that miR-617 is one of the downregulated miRNAs in lung adenocarcinoma samples compared to squamous cell carcinoma samples and is also downregulated in recurrence *vs* no-recurrence case groups of stage I non-small-cell lung carcinoma (NSCLC) (12). The above microRNA microarray analyses thus revealed that miR-617 is found to be downregulated in most cancer types or associated with chemoresistance or upregulated in a few cancers. Similarly, in oral cancer, two independent microRNA microarray analyses by Lajer et al. (13) and Rentoft et al. (14) showed that miR-617 is significantly downregulated in OSCC samples compared to normal oral samples. Moreover, Zhao and Liu (15) showed that miR-617 expression is reduced in OSCC tissues compared to their matched normal tissues and displayed a negative association with the clinical TNM stages of OSCC patients. They also showed that miR-617 partly downregulates the proto-oncogene *SERPINE1* to inhibit the progression of OSCC. But, except for *SERPINE1*, there is no other known downstream gene target of miR-617 in OSCC (15). Altogether, miR-617 is found to be consistently downregulated in OSCC tissues compared to normal oral tissues. This prompted us to further explore the mechanism of its downregulation and decipher its role in OSCC during the present study.

## 2 Materials and methods

### 2.1 Plasmid constructs

The functional features of all the constructs generated during the present study are summarized in **Supplementary Table S1**. The wild-type constructs (e.g., pmiR-617, p3’UTR-S, pDDX27, pGL3-PrMIR617-F1, pGL3-PrMIR617-F2, and pGL3-PrDDX27) were generated using human genomic DNA or cDNA as template and gene specific primers (**Supplementary Table S2**) by standard laboratory protocols. The constructs harboring mutations (pmiR-617-M and pGL3-PrDDX27-M) were generated by site-directed mutagenesis using specific primers (**Supplementary Table S3**), according to Sambrook et al. (16). pPr-null-DDX27 was generated by excising the *luc* ORF from the promoter-null pGL3-Basic vector and replacing it with the *DDX27* ORF. Thereafter, the wild-type and target site mutated *DDX27* promoter fragments were cloned upstream to *DDX27* ORF in pPr-null-DDX27 to generate pPr-DDX27 and pPr-M-DDX27 respectively. pDDX27–3’UTR was generated by cloning 3’UTR of *DDX27* downstream to pDDX27 using specific primers (**Supplementary Table S2**). All the constructs were sequenced on an ABI PRISM A310-automated sequencer (Thermo Fisher Scientific, Waltham, MA) to confirm directionality and error-free sequence of the inserts.

### 2.2 Cell culture

Human oral squamous cell carcinoma cell lines, UPCI: SCC084 and UPCI: SCC131, were a kind gift from Prof. Susanne Gollin, University of Pittsburgh, Pittsburgh, PA (17). These cell lines were generated following Institutional Review Board guidelines from consenting patients undergoing surgery for squamous cell carcinoma of the oral cavity at the University of Pittsburgh Medical Centre (17). SCC131 cells were derived from a T_2_N_2b_M_0_ lesion on the floor of the mouth of a 73-year-old Caucasian male, and SCC084 cells were derived from a T_2_N_2_M_0_ lesion of the retromolar trigone of a 52-year-old Caucasian male (17). Cells were cultured in DMEM supplemented with 10% FBS and 1X Antibiotic Antimycotic solution (Sigma-Aldrich, St. Louis, MO) at 37°C in 5% CO_2_.

### 2.3 Transient transfection

SCC131 or SCC084 cells were seeded at a density of 2 × 10^6^ cells/well in a 6-well plate and transiently transfected with an appropriate construct or a combination of constructs using the Lipofectamine 2000 Transfection Reagent (Thermo Fisher Scientific, Waltham, MA). After 48 h, cells were harvested for either total RNA isolation using TRI-Reagent or total protein lysate preparation using the CelLytic M Cell Lysis Reagent (Sigma-Aldrich, St. Louis, MO).

### 2.4 Dual-luciferase reporter assay

For the dual-luciferase reporter assay, 5 × 10^4^ cells/well in a 24-well plate were transfected with different constructs as per experimental requirement. The assay was carried out after 48 h of transfection of SCC131 cells, using the dual-luciferase reporter assay system (Promega, Madison, WI). The pRL-TK control vector, coding for renilla luciferase, was co-transfected for normalizing the transfection efficiency. Each bar representing the relative light unit (RLU) is an average of 3 biological replicates.

### 2.5 *In silico* identification of promoters

The putative promoter sequences of *MIR617* and *DDX27* were retrieved by an *in silico* search using following promoter prediction databases: DBTSS (http://dbtss.hgc.jp), Promoter 2.0 (https://services.healthtech.dtu.dk/services/Promoter-2.0/), and PROMO (http://alggen.lsi.upc.es).

### 2.6 5-Azacytidine treatment

SCC131 cells were grown for 24 h and treated with 5-Azacytidine (Sigma-Aldrich, St. Louis, MO, USA) at a final concentration of 5 µM and the vehicle-control DMSO (Sigma-Aldrich, St. Louis, MO, USA) separately for 5 days. Total RNA, protein, and genomic DNA were isolated from 5-Azacytidine- and DMSO-treated cells for further studies.

### 2.7 Bisulphite sequencing PCR analysis

The sodium bisulphite-treated genomic DNA samples from 5-Azacytidine- and DMSO-treated cells were used as templates to amplify *MIR617* promoter with methylation-specific primers designed (**Supplementary Table S4**) according to the MethPrimer database (http://www.urogene.org/methprimer; 18). Following amplification, PCR products were ligated to the pTZ57R TA cloning vector (Thermo Fisher Scientific, Waltham, MA, USA). Ten clones from each experimental set were sequenced on an ABI PRISM A310-automated sequencer (Thermo Fisher Scientific, Waltham, MA, USA) to identify the methylation status of the CpG sites in the *MIR617* promoter in 5-Azacytidine- and DMSO-treated cells.

### 2.8 Total RNA extraction and cDNA preparation

Total RNA was isolated using TRI-Reagent (Sigma-Aldrich, St. Louis, MO), and quantitated using the NanoDrop 1000 Spectrophotometer (Thermo Fischer Scientific, Waltham, MA). First-strand cDNA was synthesized using 2 µg of total RNA and a Verso cDNA Synthesis Kit (Thermo Fischer Scientific, Waltham, MA).

### 2.9 RT-qPCR

The expression level of miR-617 was determined by RT-qPCR as suggested by Sharbati-Tehrani et al. (19). Details of the primers are given in **Supplementary Table S5**. The RT-qPCR analysis was carried out using the DyNAmo ColorFlash SYBR Green qPCR Kit in a QuantStudio3 Real-Time PCR System (Thermo Fischer Scientific, Waltham, MA). *GAPDH* and *5S rRNA* were used as normalizing controls (20). The following equation Δ*Ct*_gene_ = *Ct*_gene_-*Ct*_normalizing control,_ was used to calculate the relative expression of gene normalized to *GAPDH* or *5S rRNA*. *Ct* represents cycle threshold value. Each bar representing the relative expression of a gene of interest normalized to its appropriate house-keeping gene is an average of 2 technical replicates.

### 2.10 Western hybridization

Proteins were resolved on an SDS-PAGE and then transferred to a PVDF membrane (Pall Corp., Port Washington, NY). The signal was visualized using an appropriate antibody and the Immobilon Western Chemiluminescent HRP substrate (Milipore, Billerica, MA). The following antibodies were used in this study: anti-DNMT1 antibody (1:5,000 dilution, cat# NB100–56519; Novus Biologicals, Centennial, CO, USA), anti-DDX27 antibody (1:1,000 dilution; cat# ab177950; Abcam, Cambridge, MA, USA), anti-mouse β-actin antibody (1:10,000 dilution; cat# A5441; Sigma-Aldrich, St. Louis, MO, USA), anti-phospho-p70 S6 Kinase (Thr421/Ser424) antibody (1:1,000 dilution; cat# 9204; Cell Signaling Technology, Danvers, MA, USA) and anti-p70 S6 Kinase antibody (1: 1,000 dilution; cat# 9202; Cell Signaling Technology, Danvers, MA, USA). The anti-mouse HRP-conjugated secondary antibody (cat# HP06) and anti-rabbit HRP-conjugated secondary antibody (cat# HP03) were purchased from Bangalore Genei, Bangalore, India.

### 2.11 *In silico* identification of gene targets

To predict the gene targets of miR-617, we used a consensus approach by employing five different mRNA target prediction algorithms like TargetScanHuman 8.0 (https://www.targetscan.org/vert_80/), miRDB (https://mirdb.org/), MiRanda (https://cbio.mskcc.org/miRNA2003/miranda.html), microT-CDS (Diana Tools) (https://dianalab.e-ce.uth.gr/html/dianauniverse/index.php?r=microT_CDS), and PITA (https://genie.weizmann.ac.il/pubs/mir07/mir07_prediction.html) (**Supplementary Table S6**).

### 2.12 OSCC patient samples

All the 36 matched normal oral tissue and OSCC patient samples used in the present study were ascertained at the Kidwai Memorial Institute of Oncology (KMIO), Bangalore, Karnataka. The samples were obtained as surgically resected tissues from oral cancerous lesions and adjacent normal tissues (taken from the farthest margin of surgical resection) in RNALater™ (Sigma-Aldrich, St Louis, MO, USA) and transferred to -80°C until further use. The tumors were staged according to the UICC’s (union for international cancer control) TNM (tumor-node-metastasis) classification (21). The details of the clinicopathological parameters obtained from the patients are summarized in **Supplementary Tables S7, S8**.

### 2.13 Cell viability

The total viable cell count was assessed by employing trypan blue dye exclusion assay as described in Karimi et al. (22). In brief, 24 h post transfection, OSCC cells were trypsinized and re-seeded at a density of 3 × 10^4^ cells/well in triplicates for each transfection experiment in 24-well plates. At each time point, cells from each well were trypsinized and pelleted by centrifugation and resuspended in complete DMEM. The cell suspension was mixed with an equal volume of 0.4% trypan blue dye (Sigma-Aldrich, USA) prepared in 1X PBS. For cell counting, 10 µL aliquot of the stained cells was added to a hemocytometer. The number of viable cells/mL was calculated using the following formula: number of viable cells/mL= mean of the total number of cells counted in the four quadrants (unstained cells) X dilution factor X 10^4^. Each data point representing the total viable cell count is an average of 3 technical replicates.

### 2.14 Cell proliferation

The rate of cell proliferation was determined by the CHEMICON BrdU Cell Proliferation Assay Kit (Millipore Corporation, Billerica, MA) (20). BrdU cell proliferation assay is used for the *in vitro* quantitative detection of newly synthesized DNA of actively proliferating cells. In brief, 2 × 10^3^ cells/well were seeded in triplicates in 96-well plates and transiently transfected with different plasmid constructs. The BrdU label was added on days 1, 2, and 3, and incubated for 6 h in a humidified CO_2_ incubator. The remaining protocol was followed as per the manufacturer’s instructions. The absorbance was measured at 450 nm using Infinite 200 PRO Plate reader (Tecan Group Ltd, Mannedorf, Switzerland). Each bar representing absorbance at 450 nm is an average of 3 biological replicates.

### 2.15 Coefficient of interaction

Synergism between two drugs is calculated by CDI (coefficient of drug interaction); a CDI <1 indicates a synergistic relationship (23). In the present study, we employed the CDI formula to calculate the synergistic effect of the interaction between miR-617 and *DDX27* on cell viability by co-transfecting their overexpression constructs in SCC131 cells. For our analysis, we designated it as the coefficient of interaction (CI). The CI was calculated as follows. CI = AB/(A × B), where AB is the ratio of the viable cell count post 72 h of cells co-transfected with overexpression constructs of miR-617 (pmiR-617) and *DDX27* (pPr-DDX27) to the viable cell count post 72 h of cells co-transfected with pPr-null-DDX27 (a promoter-less construct with *DDX27* ORF) and pcDNA3-EGFP, and A or B is the ratio of the viable cell count post 72 h of cells transfected with pmiR-617 or pPr-DDX27 to the viable cell count post 72 h of cells co-transfected with the pPr-null-DDX27 and pcDNA3-EGFP. Each data point representing the total viable cell count is an average of 3 technical replicates.

### 2.16 Apoptosis

The CaspGLOW Fluorescein Active Caspase-3 Staining Kit (Biovision, Mountain View, CA) was used to quantify the apoptosis of cells transfected with the appropriate constructs (24). After transfection, FITC-DEVD label was added to cells, and the rest of the steps were followed as per the manufacturer’s protocol. The fluorescence intensity was measured, using an Infinite 200 PRO plate reader (Tecan Group Ltd, Mannedorf, Switzerland). Each bar representing the caspase-3 activity is an average of 4 technical replicates.

### 2.17 Anchorage independent growth of colonies

The ability of cells to grow in an anchorage independent manner was assessed by the soft agar colony-formation assay (25). After transfecting cells with appropriate constructs, they were harvested and 7 × 10^3^ cells were plated in 1 mL of 0.35% Noble Agar (Difco, Mumbai, India) diluted with culture media in a 35 mm dish. After 14 days, colonies were imaged using the Leica Inverted Microscope Dmi1 (Leica Microsystems, Wetzlar, Germany) and counted by the OpenCFU software (https://opencfu.sourceforge.net/). Each bar representing the number of colonies per microscopic field is an average of 4 technical replicates.

### 2.18 *In vivo* assay for tumor growth

To investigate the effect of miR-617-mediated targeting of *DDX27* on tumor growth, 2×10^6^ SCC131 cells were transfected with 400 nM of a synthetic miR-617 mimic or 400 nM of a mimic control separately. After 24 h of transfection, cells from both groups were suspended separately in 200 μL of 1:1 dilution of DPBS and matrigel [Corning^®^ Matrigel^®^ Growth Factor Reduced (GFR) Basement Membrane Matrix, LDEV-free, cat # 354230, New York, USA]. A total of six 4–6 weeks old female mice (NU/J (athymic nude), three per group, were injected with either miR-617 mimic or mimic control transfected cells on both flanks. Tumor growth was monitored by measuring its volume using a digital caliper every 2 days until 27 days. The following equation was used to measure the tumor volume *V* = (*W* ^2^_X_ *L*)/2, where *L* and *W* represent length and width, respectively (20). Excised tumors were weighed at the end of 27 days. All mice were maintained on a 12:12 h light/dark cycle, in proper cages with sufficient food and water. The miRIDIAN microRNA hsa-miR-617-Mimic (cat# C-300943–01-0020) and miRIDIAN microRNA Mimic Negative Control #1 (cat # CN-001000–01-20) were purchased from Dharmacon, Inc., Lafayette, CO, USA.

### 2.19 Statistical analysis

The statistical significance of the comparison between any two experimental data sets was calculated using the student’s t test with Welch’s correction in the GraphPad Prism 8 software (Boston, MA, USA). The statistical significance of comparisons between multiple data sets was calculated by one-way analysis of variance (ANOVA). Multiple comparisons were carried out by Dunnett’s and Sidak’s multiple comparisons tests as per experimental requirement. Comparisons among data sets were considered significant when p-values were ≤0.05 (*), <0.01 (**), <0.001 (***), <0.0001 (****) or non-significant (ns) when the p-value was >0.05.

## 3 Results

### 3.1 Downregulation of miR-617 in OSCC tissues due to its promoter hypermethylation

Tumor suppressor genes are characterized by their ability to regulate the rates of cell proliferation and programmed cell death (e.g., apoptosis, autophagy etc.) to prevent cancer. The total viable cell count in a population is a balance between the rates of cell proliferation and programmed cell death. This balance is crucial for maintaining homeostasis in multicellular organisms and is also a fundamental aspect of various physiological and pathological processes. Thus, to assess if miR-617 has tumor suppressive role in OSCC cells, pmiR-617 and the vector control (pcDNA3-EGFP) were transiently transfected separately in SCC131 and SCC084 cells, and the cell viability assay was performed by cell counting using the trypan blue dye exclusion method. The results showed that miR-617 reduces the total viable cell count of OSCC cells, indicating its tumor suppressive role in OSCC (**Supplementary Figure S1A**). Furthermore, the anti-proliferative role of miR-617 in OSCC cells was established by BrdU cell proliferation assay (**Supplementary Figure S1B**).

**Figure 1:**
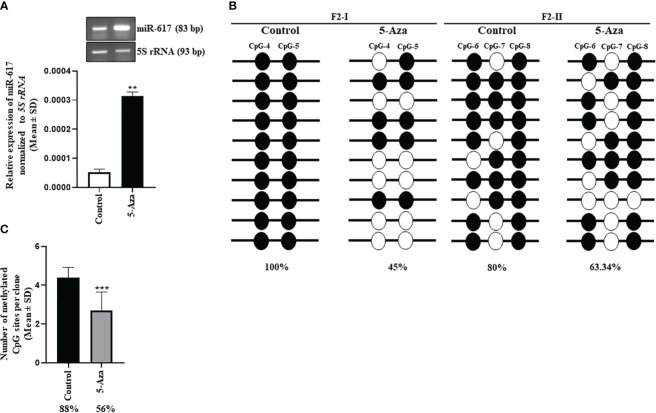
Hypermethylation of the *MIR617* promoter is one of the mechanisms for miR-617 downregulation in OSCC. **(A)** miR-617 is upregulated in 5-Azacytidine-treated SCC131 cells compared to vehicle control (DMSO)-treated cells. Representative agarose gel images to show the miR-617 upregulation in 5-Azacytidine-treated cells compared to DMSO-treated cells in semi-quantitative RT-PCR are also shown on the top. **(B)** The schematic diagram of two sub-regions F2-I and F2-II of the *MIR617* promoter chosen for BSP. BSP of F2-I fragment showed a drop in percentage methylation from 100% to 45% in 5-Azacytidine treated cells. Whereas BSP of F2-II fragment showed a drop in percentage methylation from 80% to 63.34% in 5-Azacytidine treated cells. Note, each straight line with circles represents one TA clone. **(C)** Cumulative methylation percentage for both sub-regions significantly reduces from 88% to 56% in 5-Azacytidine-treated cells compared to DMSO-treated cells. Each bar is an average of 10 TA clones. The empty and filled circles represent unmethylated and methylated CpGs respectively. 5-Aza, 5-Azacytidine. p-values were <0.01 (**) and <0.001 (***).

In cancer, one of the mechanisms for silencing/downregulation of tumor suppressor miRNAs is DNA hypermethylation at their independent promoters or host gene promoters or promoters of their transcription activators. Thus, we hypothesized that the tumor suppressor microRNAs, which are silenced by promoter DNA hypermethylation, can be reactivated by demethylating their respective promoters upon treatment of cells with the passive DNA demethylating agent 5-Azacytidine. To this end, we treated the SCC131 cells with 5-Azacytidine and DMSO separately. After validating the efficacy of the 5-Azacytidine treatment by checking the protein level of DNA methyltransferase 1 (DNMT1) using Western blot analysis and the transcript level of a positive control *MCPH1* by RT-qPCR (**Supplementary Figure S2**), we checked for the transcript level of miR-617 by miR-Q RT-qPCR and found that it was significantly reactivated in 5-Azacytidine-treated cells compared to the DMSO-treated cells (**Figure 1A**).The *MIR617* gene is located in the 4^th^ intron (34,045 bp) of the host gene *LIN7A* (**Supplementary Figure S3**). Since miRNAs located in introns of >5,000 bp length are likely to have their independent transcription units, we speculated that *MIR617* could be transcribed by an independent promoter. To characterize its promoter, we retrieved a short F1 (-365 bp to +460 bp relative to TSS) and a long F2 (-2925 bp to +250 bp relative to TSS) fragments using DBTSS, Promoter 2.0 and PROMO databases and characterized these cloned fragments (pGL3-PrMIR617-F1 and pGL3-PrMIR617-F2) for promoter activity, using the dual-luciferase reporter assay in SCC131 cells (**Supplementary Figures S4A, B, S5**). The results showed that only pGL3-PrMIR617-F2 has a significant promoter activity compared to pGL3-Basic, suggesting that the F2 fragment represents the *MIR617* promoter (**Supplementary Figure S5**). To determine the methylation status of the *MIR617* promoter post 5-Azacytidine treatment, we chose a CpG-rich region and divided it into two segments (218 bp long F2-I and 344 bp long F2-II) and performed the bisulphite sequencing PCR (BSP) analysis (**Supplementary Figure S6**). The results showed a significant decrease in cumulative methylation percentage of the *MIR617* promoter from 88% to 56% in 5-Azacytidine-treated cells compared to DMSO-treated cells (**Figures 1B, C**). This indicated that the upregulation of miR-617 following the treatment of SCC131 cells by 5-Azacytidine (**Figure 1A**) is due to hypomethylation of the *MIR617* gene promoter, further suggesting that the *MIR617* promoter hypermethylation is one of the mechanisms for miR-617 downregulation in OSCC.

### 3.2 miR-617 positively regulates DDX27 levels by interacting with its promoter

**Figure 2:**
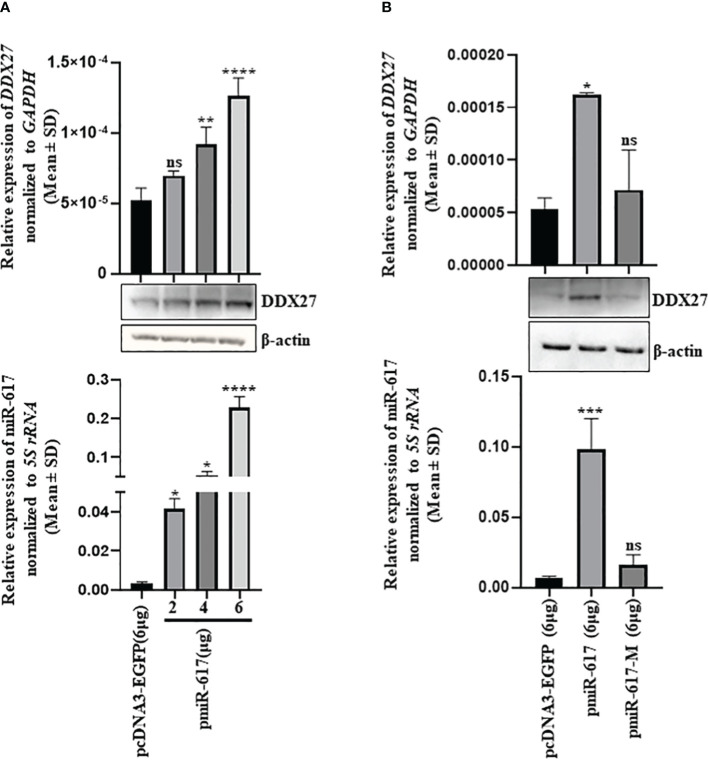
Identification of *DDX27* as a gene target for miR-617 in SCC131 cells. **(A)** A dose-dependent regulation of DDX27 transcript and protein upon transient transfection of cells with increasing doses of pmiR-617 compared to those transfected with the vector control **(B)** Upregulation of DDX27 is specific to functional miR-617 level. Transfection of the pmiR-617 construct in SCC131 cells increases the transcript and protein levels of DDX27 compared to those transfected with pmiR-617-M or the vector control. p-values were ≤0.05 (*), <0.01 (**), <0.001 (***), <0.0001 (****) and >0.05 (ns).

miR-617 demonstrated an anti-proliferative role in OSCC cells, thus we expected its gene targets to have pro-proliferative functions. We employed five miRNA target prediction tools and obtained eight putative protein-coding gene targets which have putative miR-617 binding sites in their 3’UTRs (**Supplementary Table S6**). Since *DDX27* is a known oncogene in other cancers, we chose to investigate it as a potential gene target for miR-617. To determine if miR-617 regulates *DDX27*, we transfected the vector control, pcDNA3-EGFP, and pmiR-617 in increasing quantities in SCC131 cells and observed that miR-617 upregulates DDX27 at both transcript and protein levels in a dose-dependent manner, instead of downregulating it (**Figure 2A**). To ascertain the specificity of DDX27 upregulation post miR-617 overexpression, we transfected SCC131 cells with the vector control, pmiR-617, and pmiR-617-M (miR-617 overexpression construct with the deleted seed region) separately and performed RT-qPCR and Western blot analysis. The results showed that the miR-617 level was upregulated in the cells transfected with pmiR-617 compared to those transfected with the vector control or pmiR-617-M. As expected, DDX27 level was specifically upregulated in cells transfected with pmiR-617 compared to those transfected with the vector control or pmiR-617-M (**Figure 2B**). Further, the tumor-suppressive role of miR-617 was also specific to miR-617 levels, wherein total viable cell count significantly reduced in SCC131 cells transfected with pmiR-617 in comparison to those transfected with pcDNA3-EGFP or pmiR-617-M (**Supplementary Figure S7**).

**Figure 3:**
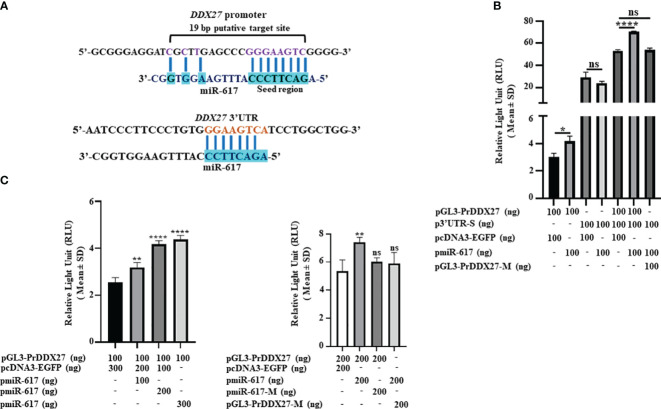
miR-617 interacts with the *DDX27* promoter. **(A)** Putative miR-617 binding sites in the promoter and 3’UTR of *DDX27*. Note, the 11 bp complementarity in the 19 bp putative target site of miR-617 in the *DDX27* promoter. **(B)** Dual-luciferase reporter assay to determine the interaction of miR-617 with 3’UTR and promoter of *DDX27* in SCC131 cells. Note, a significant increase in luciferase activity in cells co-transfected with pGL3-PrDDX27 and pmiR-617 compared to those co-transfected with pGL3-PrDDX27 and the vector control, suggesting that miR-617 interacts with the promoter of *DDX27*. Further, there was no significant change in luciferase activity in cells co-transfected with p3’UTR-S and pmiR-617 compared to those co-transfected with p3’UTR-S and the vector control, suggesting that miR-617 does not interact with the 3’UTR of *DDX27*. **(C)** miR-617 regulates the promoter activity of *DDX27* in a dose-dependent and sequence-specific manner in SCC131 cells. p-values were ≤0.05 (*), <0.01 (**), <0.0001 (****) and >0.05 (ns).

To explain the upregulation of DDX27 by miR-617, we speculated that there could be a noncanonical interaction of miR-617 with the *DDX27* promoter to upregulate it. We therefore bioinformatically retrieved the putative *DDX27* promoter fragment (-642 bp to +67 bp relative to TSS) and characterized the cloned fragment pGL3-PrDDX27 for promoter activity by dual-luciferase reporter assay in SCC131 cells (**Supplementary Figures S8A, B**). The results showed that it has a significant promoter activity compared to pGL3-Basic (**Supplementary Figure S8B**). Subsequently, we observed a putative miR-617 binding site in the *DDX27* promoter as well (**Figure 3A**). Thus, we aimed to determine if miR-617 interacts with the *DDX27* promoter or 3’UTR or both (**Figure 3B**). To this end, we transfected p3’UTR-S, pGL3-PrDDX27, and pGL3-PrDDX27-M separately or in combination with pmiR-617 in SCC131 cells and performed the dual-luciferase reporter assay. The results showed that miR-617 interacts with the *DDX27* promoter only (**Figure 3B**). Next, pGL3-PrDDX27 was co-transfected with an increasing dosage of pmiR-617 in SCC131 cells and the results showed that miR-617 positively regulates the *DDX27* promoter activity in a dose-dependent manner (**Figure 3C**). Further, co-transfection of SCC131 cells with pmiR-617 and pGL3-PrDDX27-M or pmiR-617-M and pGL3-PrDDX27 showed that miR-617 regulates the *DDX27* promoter activity in a sequence-specific manner (**Figure 3C**).

### 3.3 *In vitro* construct system recapitulates the DDX27 upregulation

**Figure 4:**
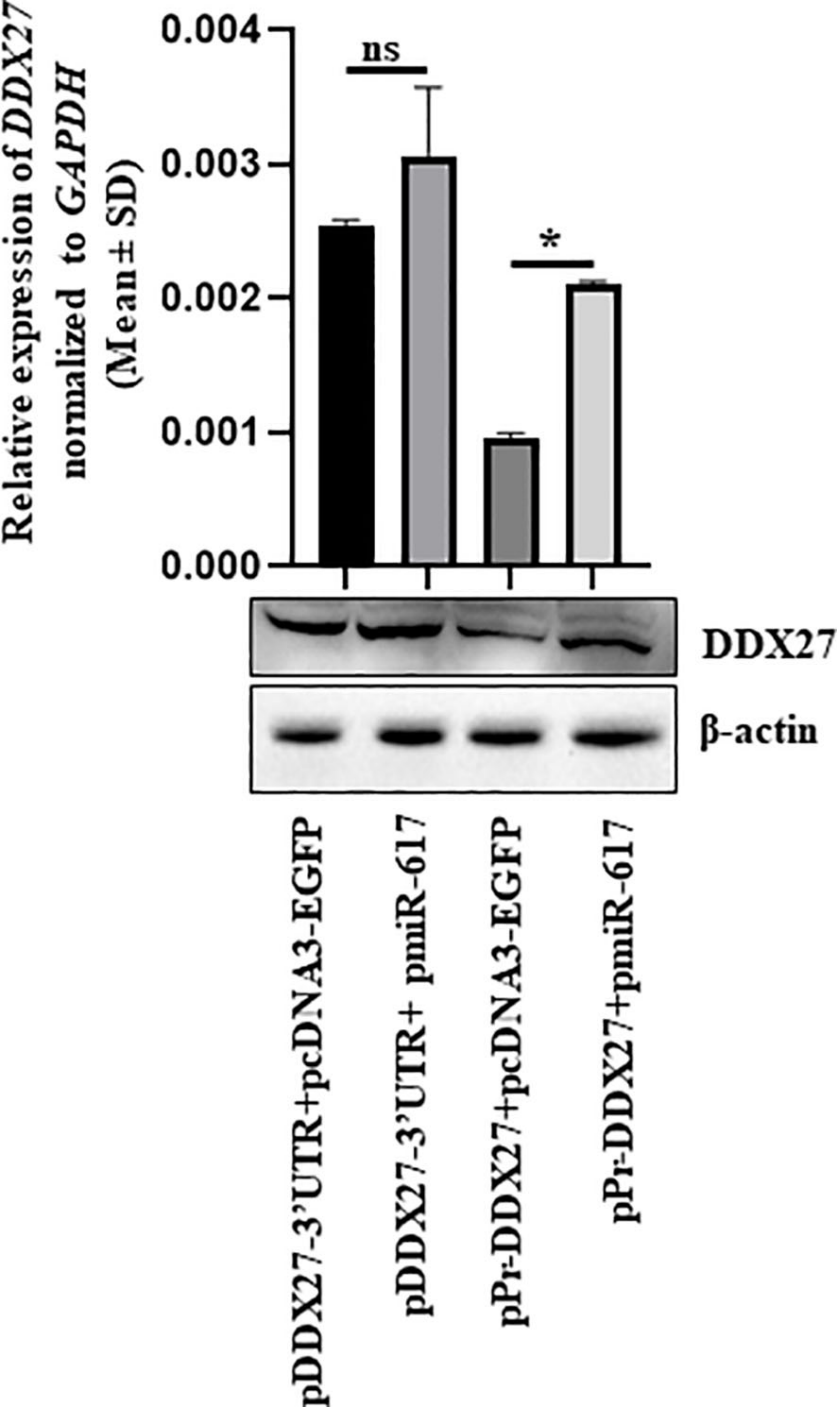
*In vitro* construct system recapitulates the DDX27 upregulation by miR-617 in SCC131 cells. Cells co-transfected with pPr-DDX27 and miR-617 show a significant induction in the levels of DDX27 transcript and protein compared to those co-transfected with pPr-DDX27 and the vector control. For each experiment, 2 μg of each plasmid construct (total 4 μg) was transfected into the cells. p-values were ≤0.05 (*) and >0.05 (ns).

A *DDX27* overexpression construct driven by its own promoter (pPr-DDX27) was generated to mimic the endogenous transcription unit (**Supplementary Figure S9**). When pPr-DDX27 was co-transfected with pmiR-617 in SCC131 cells, DDX27 was upregulated at transcript and protein levels compared to those co-transfected with pPr-DDX27 and the vector control (pcDNA3-EGFP) due to the interaction of miR-617 with the *DDX27* promoter in pPr-DDX27 (**Figure 4**). As expected, no change was observed in DDX27 transcript and protein levels in cells co-transfected with pDDX27–3’UTR and pmiR-617 compared to those co-transfected with pDDX27–3’UTR and the vector control (**Figure 4**). Thus, the *in vitro* construct system recapitulated the DDX27 upregulation observed upon miR-617 overexpression.

### 3.4 Biological relevance of the interaction of miR-617 with the *DDX27* promoter

**Figure 5:**
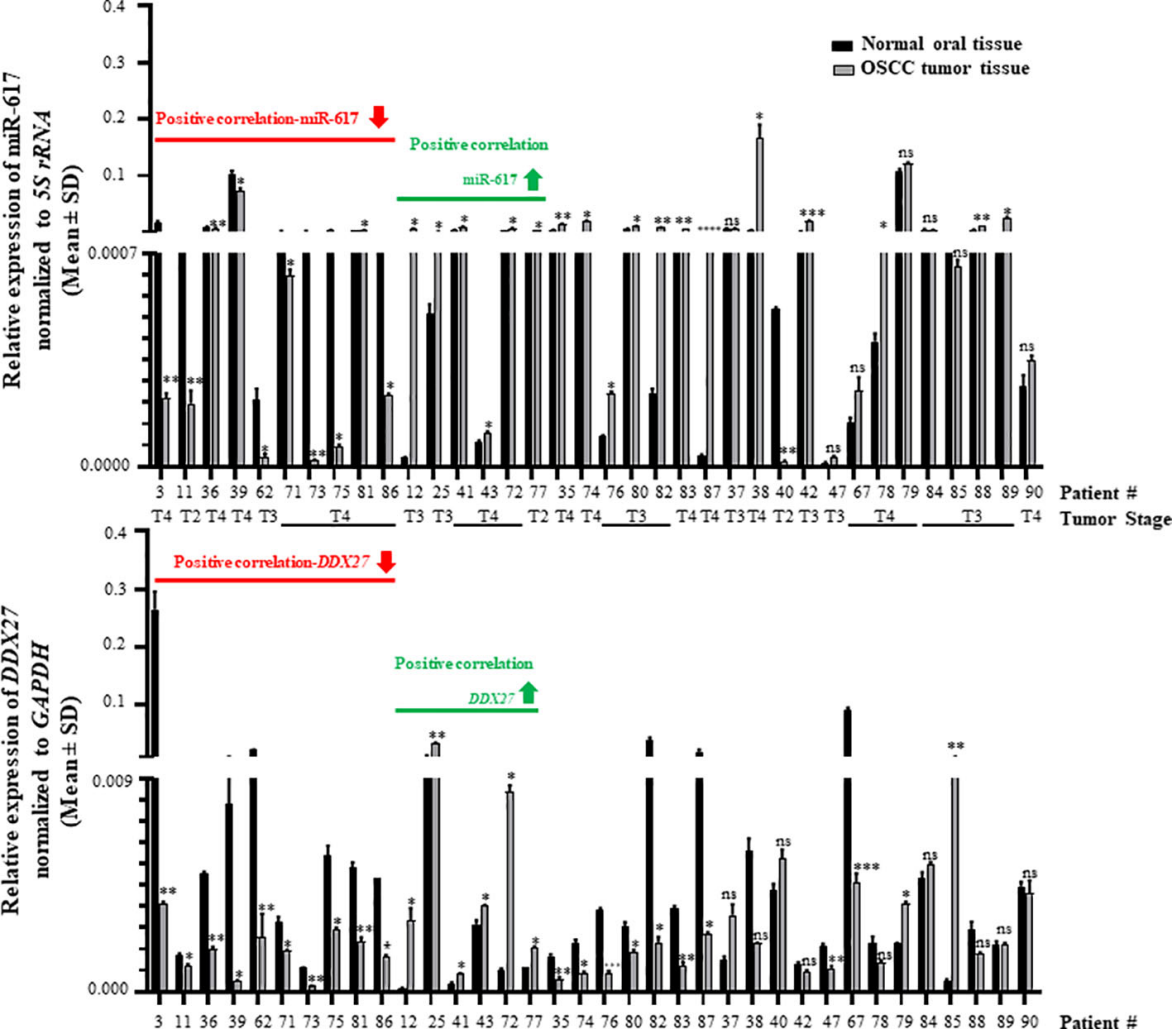
Biological relevance of the interaction between miR-617 and *DDX27* in OSCC patient samples. miR617 levels were downregulated in 11/36 OSCC samples (top panel) and *DDX27* levels were downregulated in 19/36 OSCC samples (bottom panel) compared to their matched normal oral tissue samples. Overall, 16/36 OSCC samples showed a positive correlation in the levels of miR-617 and *DDX27*. p-values were ≤0.05 (*), <0.01 (**), <0.001 (***) and >0.05 (ns).

Further, to determine the biological relevance of the interaction between miR-617 and the *DDX27* promoter, we evaluated their levels in 36 matched normal oral tissue and OSCC patient samples by RT-qPCR. The results showed that miR-617 was significantly downregulated in 11/36 (viz., patient no. 3,11,36,39,62,71,73,75,81,86, and 40) OSCC patient samples in comparison to their matched normal oral tissue samples (**Figure 5**, top panel). Alongside, *DDX27* was significantly downregulated in 19/36 (viz., patient no. 3,11,36,39,62,71,73,75,81,86,35,74,76,80,82,83,87,47, and 67) OSCC patient samples when compared to their matched normal oral tissue samples (**Figure 5**, bottom panel). Moreover, there was a significant upregulation in the levels of miR-617 in 18/36 (viz., patient no. 12,25,41,43,72,77,35,74,76,80,82,83,87,38,42,78,88, and 89) and of *DDX27* in 8/36 (viz., patient no. 12,25,41,43,72,77,79, and 85) OSCC patient samples compared to their matched normal oral tissue samples (**Figure 5**). The levels of miR-617 and *DDX27* did not show any change in 7/36 (viz., patient no. 37,47,67,79,84,85, and 90) and 9/36 (viz., patient no. 37,38,40,42,78,84,88,89, and 90) OSCC patient samples, respectively, when compared to their matched normal oral tissue samples (**Figure 5**). We observed that miR-617 and *DDX27* levels were concomitantly downregulated in 10/36 (viz., patient no. 3,11,36,39,62,71,73,75,81, and 86) and upregulated in 6/36 (viz., patient no. 12,25,41,43,72, and 77) OSCC samples compared to their matched normal oral tissue samples (**Figure 5**). Taken together, RT-qPCR analyses showed that in the OSCC tissues, miR-617 and *DDX27* levels concomitantly increased or decreased in 16/36 (44.44%) samples compared to their matched normal tissue samples (viz., patient no. 3,11,36,39,62,71,73,75,81,86,12,25,41,43,72, and 77), exemplifying the biological relevance of the interaction between miR-617 and the *DDX27* promoter (**Figure 5**). However, we did not observe a concomitant increase or decrease of miR-617 and *DDX27* levels in the remaining 20/36 (55.56%) OSCC samples compared to their matched normal oral tissue samples.

Interestingly, 11/16 (68.75%) OSCC samples which showed the biological relevance between miR-617 and *DDX27* levels are T4 stage tumor samples; 10/16 (62.5%) OSCC samples originated from OSCC of the buccal mucosa (**Figure 5**; **Supplementary Table S8**). Additionally, it is noteworthy that *DDX27* levels were downregulated in 10/11 (90.9%) OSCC samples compared to their matched normal oral tissue samples (viz., patient no. 3,11,36,39,62,71,73,75,81, and 86), wherein miR-617 levels were also downregulated (**Figure 5**). *DDX27* levels were upregulated in 6/18 (30%) OSCC samples compared to their matched normal oral tissue samples (viz., patient no. 12,25,41,43,72, and 77), wherein miR-617 levels were also upregulated.

### 3.5 Role of DDX27 in OSCC cells

**Figure 6:**
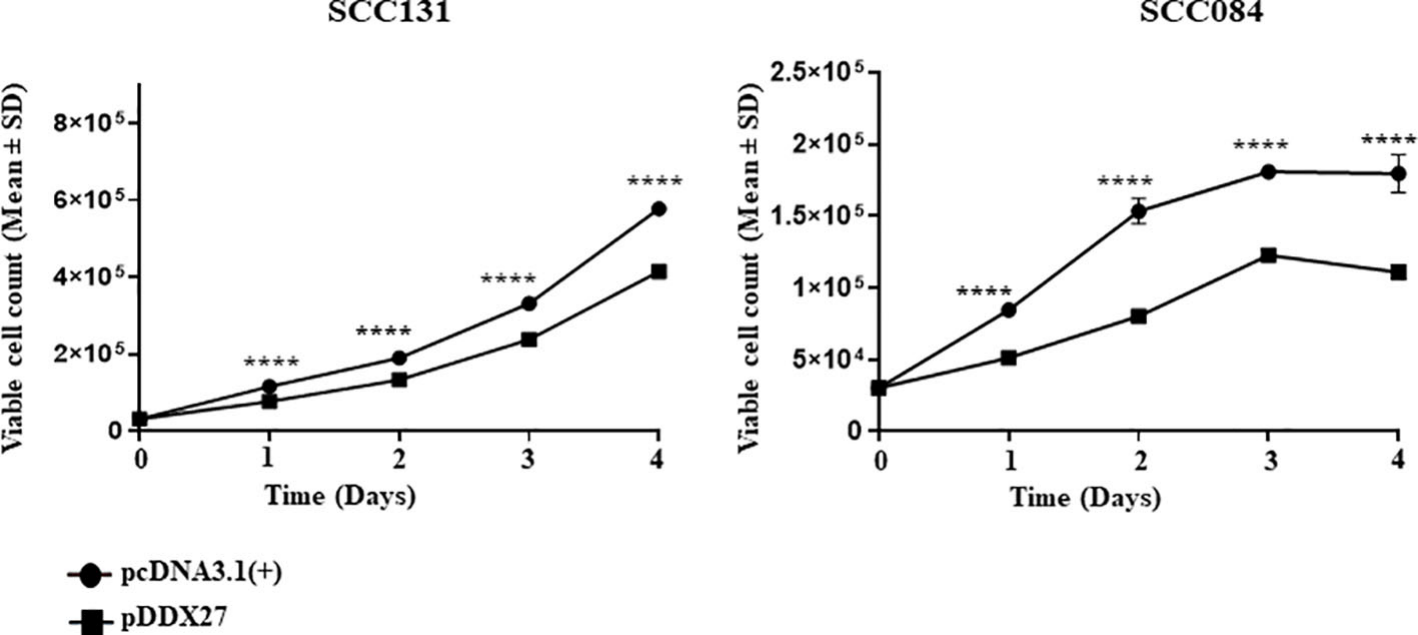
DDX27 reduces cell viability of SCC131 and SCC084 cells. Transient overexpression of DDX27 significantly decreases viability of both SCC131 and SCC084 cells compared to the vector control transfected cells. For each experiment, 6 μg of plasmid construct was transfected into the cells. p-value was <0.0001 (****).

The positive correlation in the transcript levels of miR-617 and *DDX27* served as an impetus to study the role of DDX27 in cell viability and proliferation. We transfected pDDX27 and the vector control, pcDNA3.1(+), separately in OSCC cells and found a significant reduction in total viable cell count of cells transfected with pDDX27 compared to those transfected with the vector control (**Figure 6**). Furthermore, BrdU cell proliferation assay in SCC131 cells showed that there was a significant decrease in proliferation of cells transfected with pDDX27 compared to those transfected with the vector control (**Supplementary Figure S10**). This suggested that DDX27 acts as a negative regulator of cell viability and proliferation in OSCC cells contrary to its oncogenic role in other cancers.

### 3.6 Molecular effectors of the miR-617/DDX27 axis

**Figure 7:**
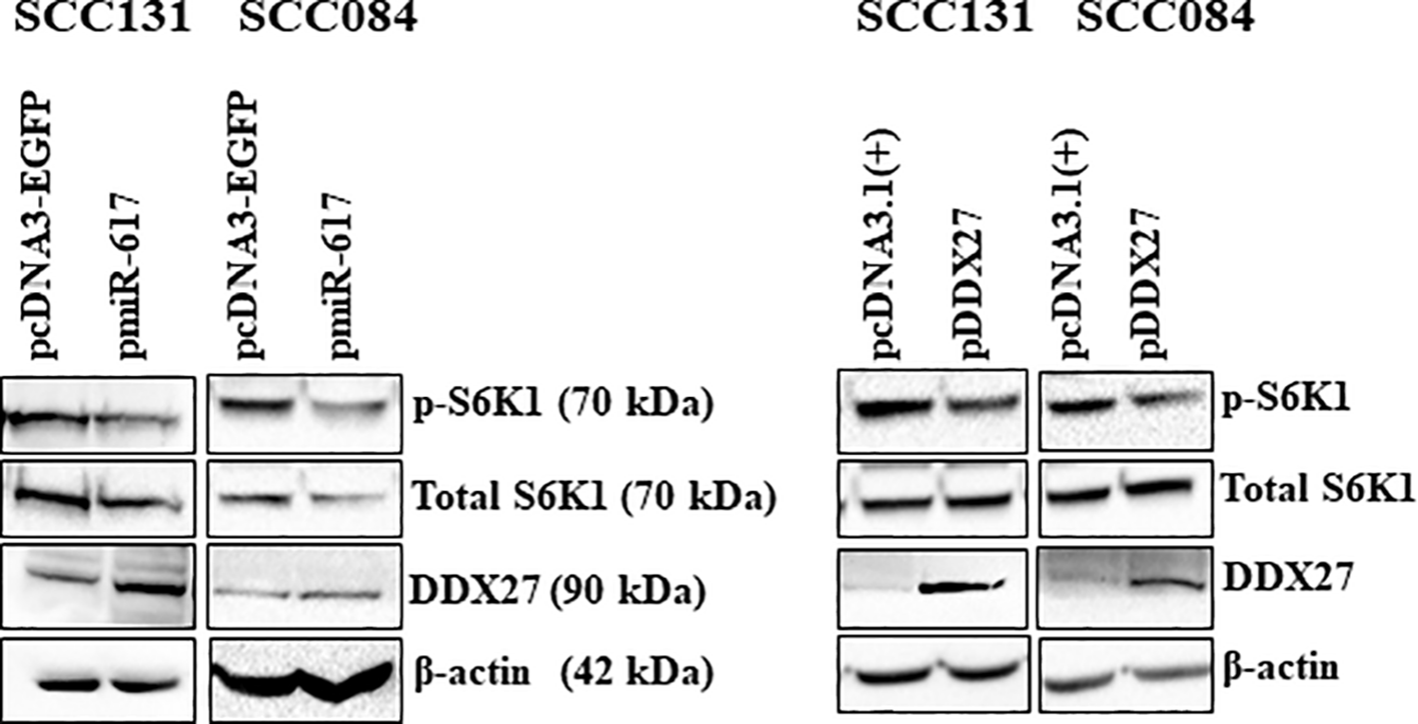
miR-617 downregulates the PI3K/AKT/MTOR pathway, in part, by regulating DDX27 in OSCC cells. Overexpression of miR-617 and DDX27 reduces p-S6K1 (phospho-S6K1) levels in OSCC cells. For each experiment, 6 μg of plasmid construct was transfected into the cells.

Next, to explore the potential molecular effectors of the miR-617/DDX27 axis, we chose to examine the PI3K/AKT/MTOR pathway as it is one of the most frequently activated pathways in cancers, including OSCC. We transfected OSCC cells with pmiR-617 or pDDX27 separately and assessed the levels of phospho-S6K1 (p-S6K1), the molecular readout of this pathway. The results showed that miR-617 and DDX27 suppress the PI3K/AKT/MTOR pathway activation by reducing p-S6K1 levels, indicating that miR-617 modulates this pathway, in part, via regulating DDX27 levels. This further correlates the reduction in cell proliferation by miR-617 to the decreased activation of the pro-survival PI3K/AKT/MTOR pathway (**Figure 7**).

### 3.7 miR-617 regulates the hallmarks of cancer, in part, by interacting with the *DDX27* promoter

We co-transfected OSCC cells with different combinations of pmiR-617, pmiR-617-M and *DDX27* constructs (viz., pPr-null-DDX27 and pPr-DDX27) to investigate the effect of miR-617 on the protein levels of DDX27. The results showed that the endogenous DDX27 level was upregulated in OSCC cells co-transfected with pPr-null-DDX27 and pmiR617 compared to those co-transfected with pPr-null-DDX27 and pcDNA3-EGFP (**Supplementary Figure S11**). Moreover, there was a further increase in DDX27 level in cells co-transfected with pPr-DDX27 and pmiR-617 in comparison to those co-transfected with pPr-DDX27 and pcDNA3-EGFP by virtue of the interaction between miR-617 and the endogenous and exogenous promoters of *DDX27* (**Supplementary Figure S11**). As expected, in OSCC cells co-transfected with pPr-DDX27 and pmiR-617-M neither the endogenous *DDX27* promoter nor the *DDX27* promoter in pPr-DDX27 was induced to cause DDX27 upregulation in comparison to those co-transfected with pPr-DDX27 and pcDNA3-EGFP, due to the absence of a functional miR-617. This data further underscores the exclusivity of DDX27 upregulation by miR-617 (**Supplementary Figure S11**).

**Figure 8:**
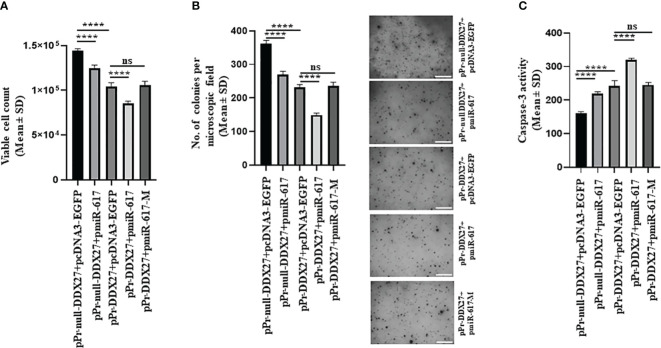
miR-617 regulates the hallmarks of cancer, in part, by targeting the promoter of *DDX27* in SCC131 cells. **(A)** miR-617 negatively regulates cell viability of cells by targeting the *DDX27* promoter. **(B)** miR-617 negatively regulates anchorage independent growth of cells by targeting the *DDX27* promoter. Representative micrographs of soft-agar colonies are shown on the right (scale bar = 500 mm). **(C)** miR-617 positively regulates cellular apoptosis by targeting the *DDX27* promoter. For each experiment, 4 μg of each plasmid construct (total 8 μg) was transfected into the cells. p-values were <0.0001 (****) and >0.05 (ns).

To investigate the effect of miR-617-mediated upregulation of DDX27 on cancer hallmarks, we co-transfected the same set of constructs in SCC131 and SCC084 cells. We demonstrated that miR-617 and DDX27 are negative regulators of cell viability and proliferation (**Supplementary Figures S1A, B, 6, S10**). Thus, as expected, cell viability and anchorage-independent growth was significantly reduced in SCC131 cells co-transfected with pPr-null-DDX27 and pmiR-617 or pPr-DDX27 and pcDNA3-EGFP compared to those co-transfected with pPr-null-DDX27 and pcDNA3-EGFP (**Figures 8A, B**). Moreover, the interaction of miR-617 with the endogenous and exogenous *DDX27* promoters further reduced cell viability and anchorage-independent growth in SCC131 cells co-transfected with pPr-DDX27 and pmiR-617 compared to cells co-transfected with pPr-DDX27 and pcDNA3-EGFP (**Figures 8A, B**). This interaction was abolished in those co-transfected with pPr-DDX27 and pmiR-617-M, indicating that miR-617 regulates cell viability and anchorage-independent growth by targeting the *DDX27* promoter in SCC131 cells (**Figures 8A, B**). Similarly, we also showed that miR-617 positively regulates apoptotic induction of SCC131 cells, in part, via targeting the promoter of *DDX27* (**Figure 8C**). Thus, our data in SCC131 cells suggested that the significant reduction in OSCC cell viability that occurs upon overexpression of miR-617 is, in part, a combined effect of reduced cell proliferation and increased apoptosis brought about by upregulation of DDX27. miR-617-mediated upregulation of DDX27 showed a similar effect on cancer hallmarks in SCC084 cells (**Supplementary Figure S12**).

### 3.8 Functional synergism between miR-617 and *DDX27*

We also showed that there is a functional synergism between miR-617 and *DDX27* by calculating their CI (co-efficient of interaction) in SCC131 cells. We observed that the CI in cells co-transfected with pPr-DDX27 and pmiR-617 was 0.83, indicating marked functional synergism (**Supplementary Figure S13**). As expected, CI was 1 (actual value 0.99) in cells co-transfected with pPr-M-DDX27 and pmiR-617. Since there is functional synergism between miR-617 and DDX27, we decided to test if there is a positive feedback loop between them as well. Thus, we transfected SCC131 cells with an increasing dosage of pDDX27 and observed that the endogenous miR-617 levels were significantly upregulated compared to those transfected with the vector control (**Supplementary Figure S14**). This suggested that DDX27, in turn, can also upregulate miR-617 at the transcriptional level and/or in its biogenesis.

### 3.9 miR-617 exerts its anti-tumorigenic potential by regulating DDX27 levels

**Figure 9:**
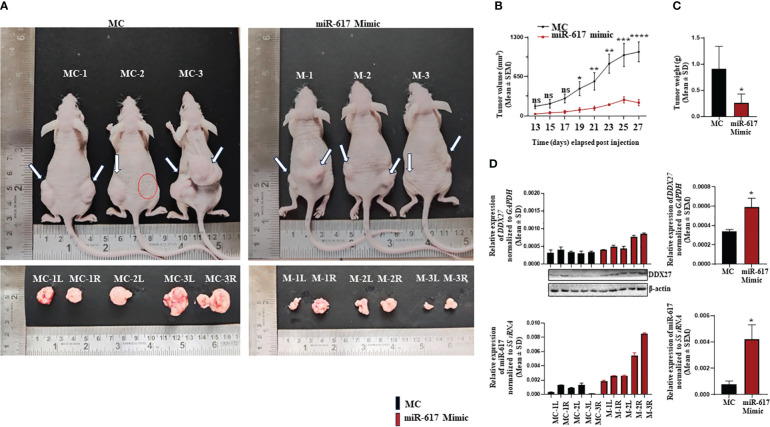
The effect of a synthetic miR-617 mimic on SCC131 cells-derived xenografts in nude mice. **(A)** Top panel: photographs of nude mice showing tumor growth on Day 27 of injection of cells pre-treated with miR-617 mimic and mimic control. Bottom panel: Excised xenografts from cells-pretreated with miR-617 mimic and mimic control on Day 27. The dotted red circle denotes the absence of tumor development. **(B)** Effect of miR-617 mimic on the volume of xenografts during a time course of 13–27 days. For mimic control group: n=5, Day 13–27 and, for miR-617 mimic group: n=4, Day 13; n=3, Day 15–21; n=4; Day 23; n=6, Day 25–27 (n=number of tumors measured). **(C)** Effect of miR-617 mimic on the weight of xenografts on Day 27. For miR-617 mimic and the mimic control groups, 3 animals were injected per group. **(D)** The levels of miR-617 and DDX27 in OSCC xenografts. L, left posterior flank; R, right posterior flank; MC, mimic control; and, M, mimic-617. p-values were ≤0.05 (*), <0.01 (**), <0.001 (***), <0.0001 (****) and >0.05 (ns).

To explore the therapeutic potential of miR-617-mediated upregulation of DDX27 on OSCC xenografts in nude mice, we transfected SCC131 cells with different quantities of a synthetic miR-617 mimic and a relevant mimic control separately to optimize the dosage. The results showed that both 200 nM and 400 nM dosages of miR-617 mimic effectively increased the levels of DDX27 in SCC131 cells in a dose-dependent manner (**Supplementary Figure S15A**). As expected, the RT-qPCR analysis showed a significantly robust expression of miR-617 and a concomitant increase in *DDX27* levels in mimic-transfected cells compared to those transfected with the mimic control, confirming its specificity (**Supplementary Figure S15A**). To further ascertain the exact dosage to perform OSCC xenograft studies, we checked for the effect of miR-617 mimic on viability of SCC131 cells. We observed that the cell viability is significantly reduced in cells transfected with 200 nM and 400 nM of miR-617 mimic in comparison to those transfected with the mimic control (**Supplementary Figure S15B**). However, a maximum reduction in cell viability was observed in cells transfected with 400 nM of miR-617 mimic, suggesting that 400 nM of miR-617 mimic is optimum to perform OSCC xenograft study in nude mice (**Supplementary Figure S15B**). We then transfected 400 nM of miR-617 mimic or mimic control in SCC131 cells for the nude mice experiments. The results showed that nude mice xenografts derived from SCC131 cells pre-transfected with 400 nM of the miR-617 mimic showed a significant reduction in tumor volume and weight compared to those derived from SCC131 cells pre-transfected with 400 nM of the mimic control (**Figures 9A–C**). As expected, subsequent RT-qPCR and Western blot analysis showed that DDX27 was significantly upregulated in OSCC xenografts with the miR-617 mimic in comparison to those with the mimic control (**Figure 9D**). Therefore, these results indicated that miR-617 acts as a tumor suppressor in OSCC, in part, by inducing DDX27 levels.

## 4 Discussion

The present study demonstrates that the miR-617 reduces viability and proliferation of OSCC cells, indicating its anti-proliferative function (**Supplementary Figures S1A, B**). A recent study by Zhao and Liu (15) also corroborates our finding wherein ectopic expression of miR-617 mimic in OSCC cells (SCC-090-HPV positive and PE/CA-PJ41-HPV negative) led to a significant reduction in cell proliferation. In a study by Venturelli et al. (26), microRNA expression chip analysis showed that miR-617 level was significantly upregulated in metastatic melanoma cells treated with 8 mM pharmacological dosage of ascorbate (inhibitor of DNMT) which is in congruence with the transcriptional reactivation of miR-617 post 5-Azacytidine treatment of SCC131 cells in the present study (**Figure 1A**). The bisulphite sequencing PCR analysis showed a significant decrease in cumulative methylation percentage of the *MIR617* promoter from 88% to 56% in 5-Azacytidine-treated cells compared to DMSO-treated cells (**Figures 1B, C**), indicating that the promoter hypermethylation could be one of the silencing mechanisms responsible for miR-617 downregulation in OSCC as reported in the literature (13–15).

Contrary to our expectation, DDX27 was upregulated upon overexpression of miR-617 in SCC131 cells, both at the transcript and protein levels, instead of showing a presumed downregulation (**Figure 2A**). This data indicated some non-canonical mode of miRNA action at play. MiRNAs are known to interact with 3’UTR and 5’UTR of cognate mRNAs and the promoter of genes to cause their upregulation (27–29). The present results showed that miR-617 interacts with the *DDX27* promoter only and regulates its activity in a dose-dependent and sequence-specific manner (**Figures 3B, C**). This phenomenon can be attributed to a biologically irrelevant interaction between miR-617 and the *DDX27* 3’UTR or a biased localization of miR-617 to the cell nucleus, thereby limiting cytoplasmic functions. Moreover, according to the miRNALoc server (http://cabgrid.res.in:8080/mirnaloc/) prediction, miR-617 has a greater propensity to localize to the nucleus over the cell cytoplasm, further implying its nuclear function. Generally, instances of transcriptional gene activation (TGA) by miRNAs have been associated with an increase in target gene expression by targeting their promoter regions (30–40) (**Supplementary Table S9**). The first ever study of TGA by miRNAs dates to 2008, when Place et al. (29) showed that ectopic expression of miR-373 in prostate cancer cells induced the levels of E-cadherin (*CDH1*) and cold shock domain containing C2 (*CSCD2*) by interacting with the reverse complementary sequences in their promoters. Another report from the same group demonstrated that, in prostate cancer cells, ectopic expression of miR-205 increased the *IL35* and *IL24* levels by binding to complementary sequences in their promoters, accompanied by enrichment of RNA polymerase II and histone marks associated with active transcription (41).

A positive correlation in the levels of miR-617 and *DDX27* was observed in 16/36 (44.44%) OSCC samples compared to their matched normal tissues (**Figure 5**), exemplifying the biological relevance of their interaction. However, we did not observe a concomitant increase or decrease in miR-617 and *DDX27* levels in 20/36 (55.56%) OSCC samples compared to their matched normal oral tissue samples. miRNA levels remained unchanged in 7/36 OSCC samples compared to their matched normal oral tissue samples (viz., patient no. 37,47,67,79,84,85, and 90). In these patient samples, *DDX27* levels were either downregulated, upregulated, or unchanged in the tumor tissues compared to the paired normal oral tissue samples (**Figure 5**). The discordance in miR-617 and *DDX27* levels can be attributed to the involvement of additional molecular players such as intratumoral heterogeneity, variable etiopathogenesis, and heterogeneous genetic composition of each patient (24, 42, 43). A possible explanation for the intratumoral heterogeneity in miRNA expression could be variations in the cellular composition of tumor samples. The presence of different tumor cell clones would be another potential explanation for heterogeneity in miRNA expression (44). Besides, miR-617 is one of the many molecular regulators of *DDX27*. Thus, the *DDX27* level in a particular patient is an outcome of the combined influence of all its molecular regulators, amenable to intratumoral heterogeneity. Up until now, the biological relevance of this miRNA-target gene pair was unexplored in cancer and this study is the first of its kind to explore the biological relevance of a miRNA-target gene pair in context to TGA by miRNAs in oral cancer. Remarkably, a pan-cancer analysis with 8,375 patient samples across 31 major human cancers from the TCGA (The Cancer Genome Atlas) database showed that the positive miRNA-gene correlations are surprisingly prevalent and consistent across cancer types and show distinct patterns than negative correlations (45). Till date, increased DDX27 levels have been correlated with poor prognosis and outcome in gastric, breast, colorectal, and hepatocellular cancer patients, indicating a positive association with carcinogenesis (46–49). However, we found that 19/36 (52.77%) OSCC samples showed significant reduction in *DDX27* levels compared to their matched normal samples (**Figure 5**). Contrary to its oncogenic role in other cancers, cell viability and proliferation assay in OSCC cells surprisingly showed a reduction in total viable cell count and proliferation upon DDX27 overexpression (**Figure 6**; **Supplementary Figure S10**), thereby suggesting an anti-proliferative role of DDX27 for the first time in OSCC cells. Interestingly, DEAD-box proteins have dual roles in cancer progression and the precise role of a DEAD-box protein depends on their interacting partners, the expression and/or function of which may also be independently altered during cancer development (50). For example, *DDX3*, *DDX5* and *DDX17* act as oncogenes or tumor-suppressors in a context-dependent manner (50). This is the first time that *DDX27* has been associated with reduced cell viability and proliferation in OSCC cell lines and decreased levels in OSCC tumors in comparison to the matched normal tissues.

We have also demonstrated that miR-617 negatively regulates cell proliferation and anchorage-independent growth and positively modulates apoptosis by regulating DDX27 levels (**Figures 8A–C**). This is in line with a recent study wherein they established miR-617 as a negative regulator of cell proliferation and a positive modulator of apoptosis in OSCC cells (15). The functional synergism between miR-617 and DDX27 provided us with an impetus to explore any possible feedback mechanism between them to regulate each other. There is an instance of miR-200 family demonstrating a negative feedback loop with its gene targets (e.g., *ZEB1* and *ZEB2*) (51, 52). DDX27 belongs to a family of putative RNA helicases implicated in a number of cellular processes involving alteration of RNA secondary structure. Hou et al. (32) showed that the plant DEAD-box RNA helicase 27 (RH27) was associated with the biogenesis of miRNAs. Thus, we wanted to explore if DDX27 regulates miR-617 level. We have already established that miR-617 upregulates DDX27, and our preliminary data suggests that, in turn, DDX27 can also upregulate miR-617 either at the transcriptional level (**Supplementary Figure S14**) and/or in its biogenesis, thereby indicating a positive feedback loop.

## 5 Conclusion

**Figure 10:**
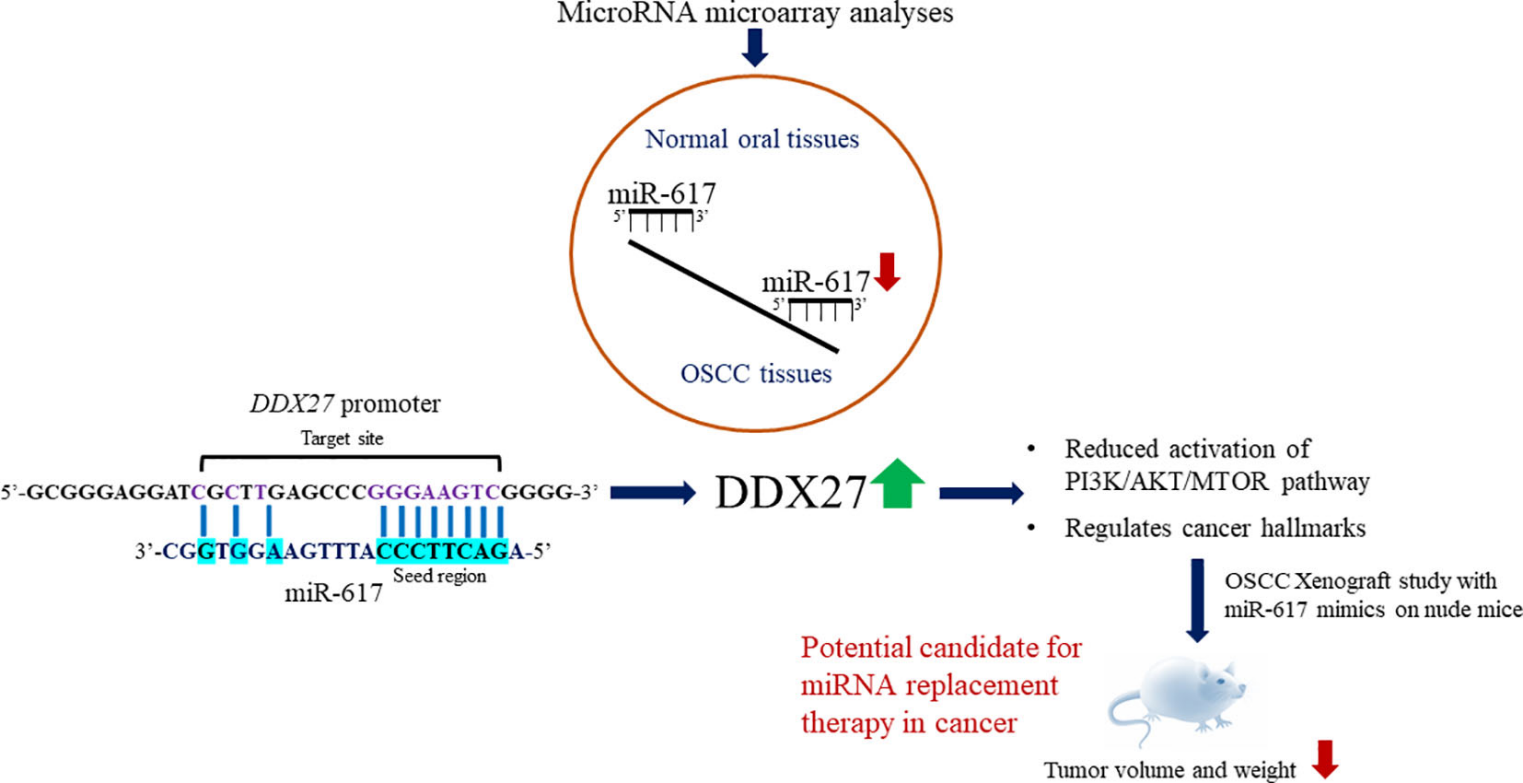
Summary of the study. Key findings of the study are outlined here.

Our *in vitro* studies have established the anti-tumorigenic activity of miR-617 via targeting the promoter of *DDX27*. The downregulation of miR-617 in OSCC is due to hypermethylation of the *MIR617* promoter. miR-617 regulates cell proliferation, anchorage-independent growth, and apoptosis of OSCC cells by modulating DDX27 levels. Further, our pre-clinical *in vivo* nude mice study indicates that synthetic miR-617 mimics can be potential candidates for miRNA replacement therapy in OSCC and perhaps in other cancers (**Figure 10**).

## Data availability statement

The original contributions presented in the study are included in the article/**Supplementary Material**. Further inquiries can be directed to the corresponding authors.

## Ethics statement

The studies involving humans were approved by the ethics committee of KMIO, Bangalore (approval # KMIO/MEC/021/05.January.2018). The studies were conducted in accordance with the local legislation and institutional requirements. The participants provided their written informed consent to participate in this study. The animal study was approved by the animal ethics committee (approval # CAF/Ethics/937/2023) of the Indian Institute of Science, Bangalore. The study was conducted in accordance with the local legislation and institutional requirements.

## Author contributions

NS: Writing – original draft, Methodology, Investigation, Formal analysis, Data curation, Conceptualization, Writing – review & editing. RM: Writing – review & editing, Resources. CG: Writing – review & editing, Resources. AK: Writing – review & editing, Supervision, Funding acquisition, Conceptualization.
