# Supplementary material for: miR-617 interacts with the promoter of DDX27 and positively regulates its expression: implications for cancer therapeutics
Source: Front Oncol. 2024 Jun 13;14:1411539. doi: 10.3389/fonc.2024.1411539 (PMC11208480; doi:10.3389/fonc.2024.1411539)
Supplement: Supplementary file 1 [file Presentation_1.pptx]

## Slide 1
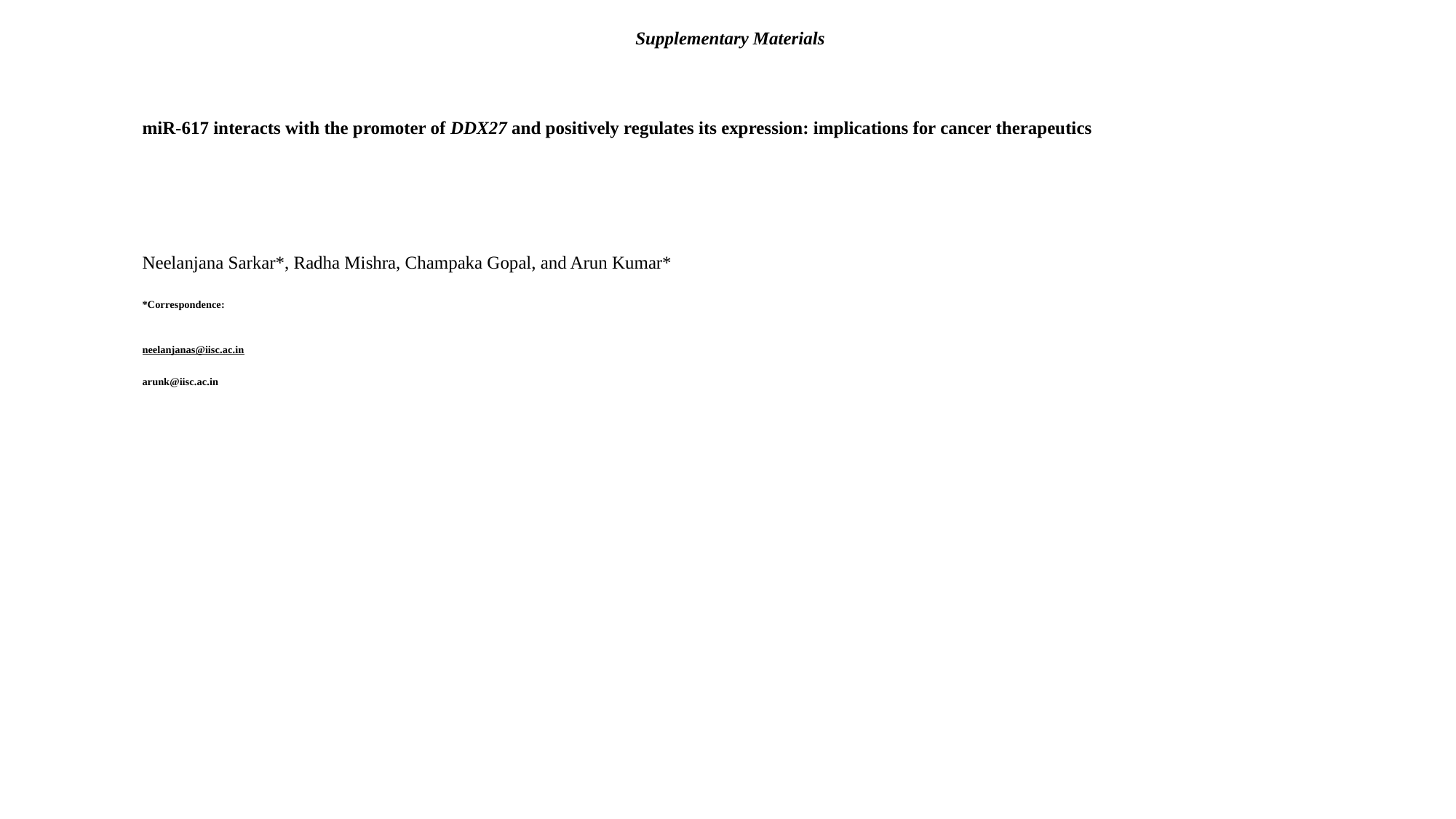

Supplementary Materials
miR-617 interacts with the promoter of DDX27 and positively regulates its expression: implications for cancer therapeutics
Neelanjana Sarkar*, Radha Mishra, Champaka Gopal, and Arun Kumar*
*Correspondence:
neelanjanas@iisc.ac.in
arunk@iisc.ac.in

## Slide 2
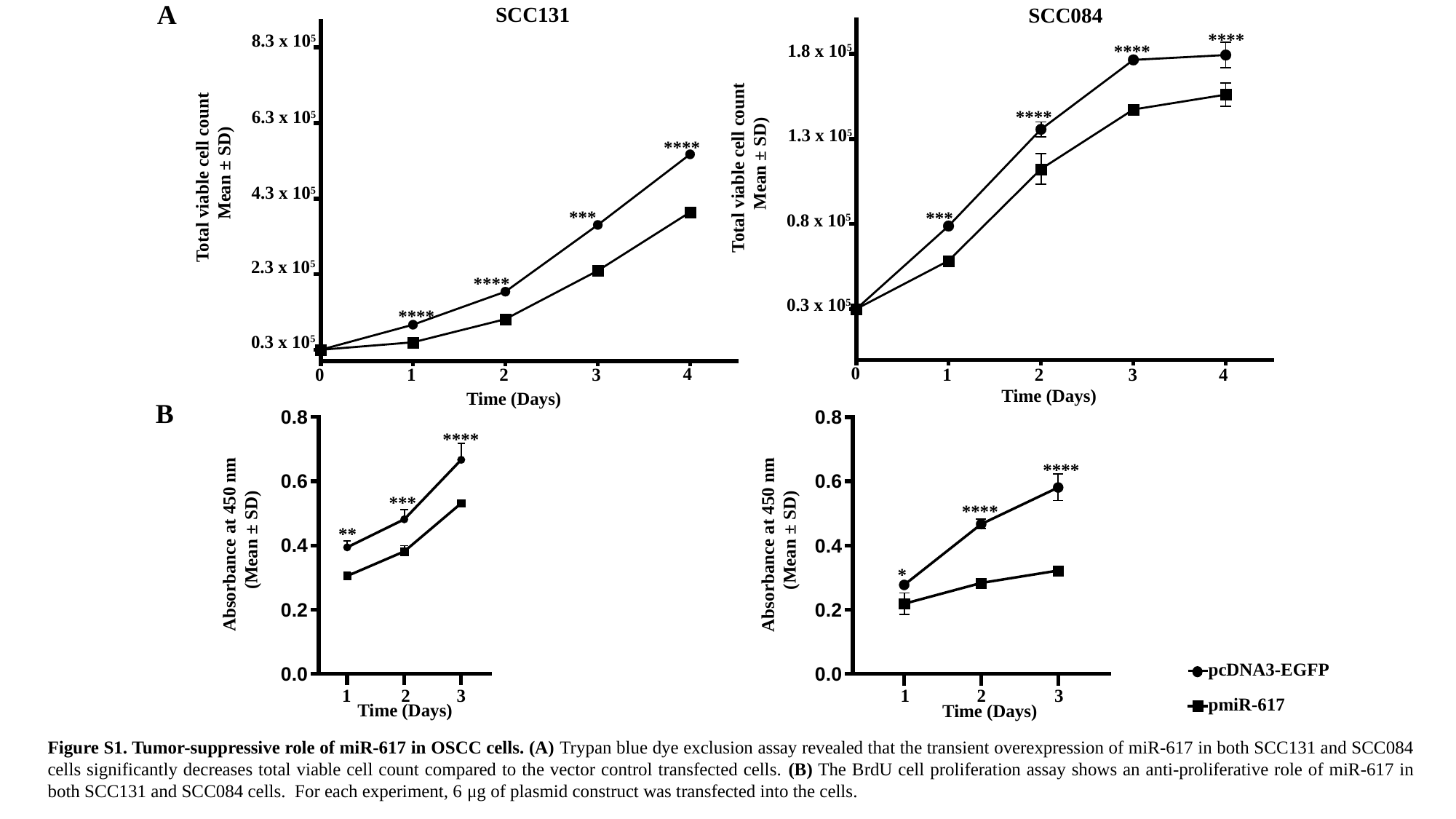

Total viable cell count
Mean ± SD)
SCC084
1.8 x 10^5^
****
****
1.3 x 10^5^
****
0.8 x 10^5^
***
Time (Days)
0.3 x 10^5^
0
1
3
4
2
SCC131
Total viable cell count
Mean ± SD)
****
***
****
****
Time (Days)
4
1
3
2
0
8.3 x 10^5^
6.3 x 10^5^
4.3 x 10^5^
2.3 x 10^5^
0.3 x 10^5^
A
Absorbance at 450 nm
 (Mean ± SD)
1
3
2
****
***
**
Time (Days)
Absorbance at 450 nm
 (Mean ± SD)
****
****
Time (Days)
*
1
3
2
B
pcDNA3-EGFP
pmiR-617
Figure S1. Tumor-suppressive role of miR-617 in OSCC cells. (A) Trypan blue dye exclusion assay revealed that the transient overexpression of miR-617 in both SCC131 and SCC084 cells significantly decreases total viable cell count compared to the vector control transfected cells. (B) The BrdU cell proliferation assay shows an anti-proliferative role of miR-617 in both SCC131 and SCC084 cells. For each experiment, 6 μg of plasmid construct was transfected into the cells.

## Slide 3
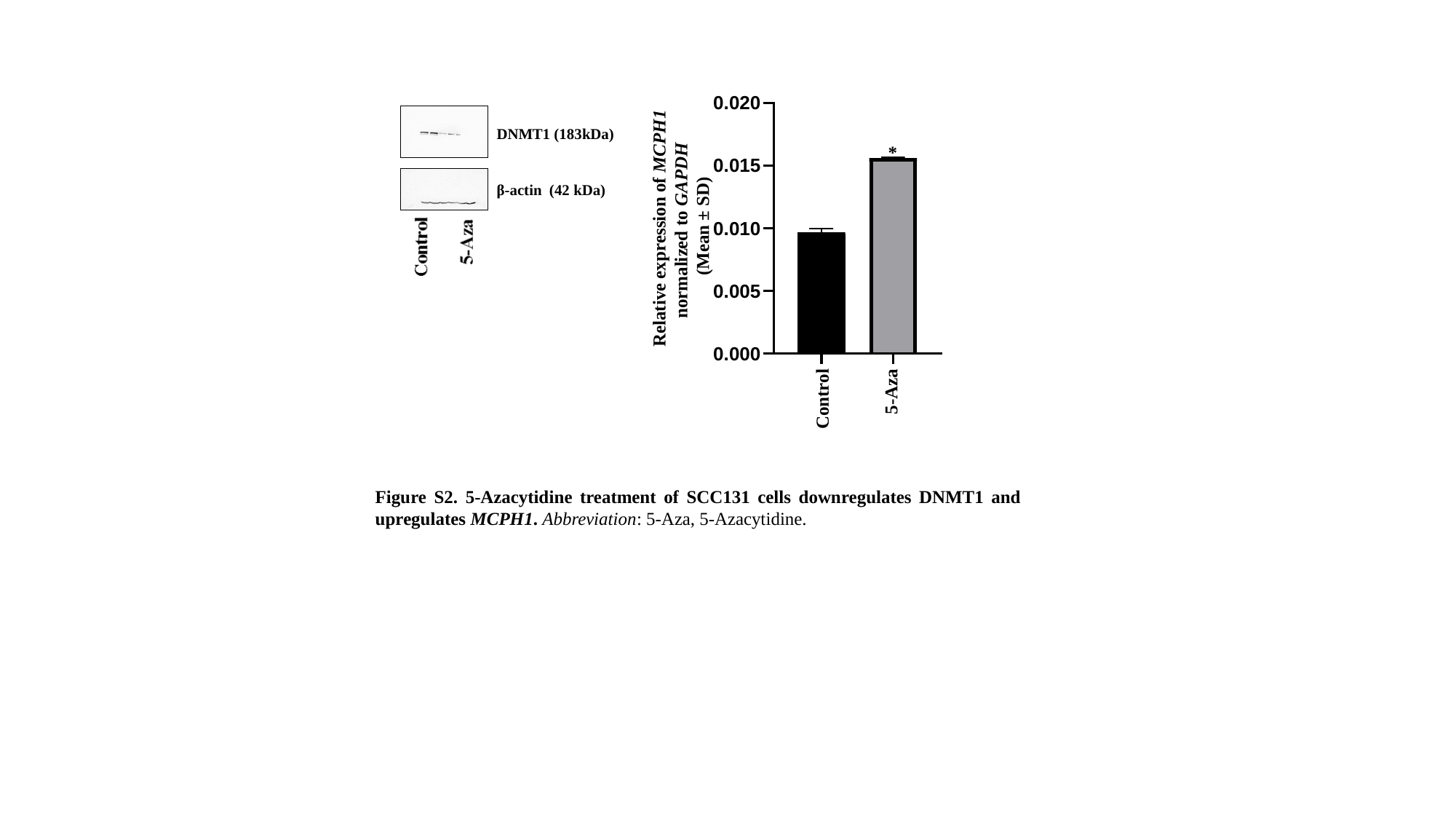

Relative expression of MCPH1 normalized to GAPDH
 (Mean ± SD)
 5-Aza
Control
*
DNMT1 (183kDa)
β-actin (42 kDa)
Figure S2. 5-Azacytidine treatment of SCC131 cells downregulates DNMT1 and upregulates MCPH1. Abbreviation: 5-Aza, 5-Azacytidine.

## Slide 4
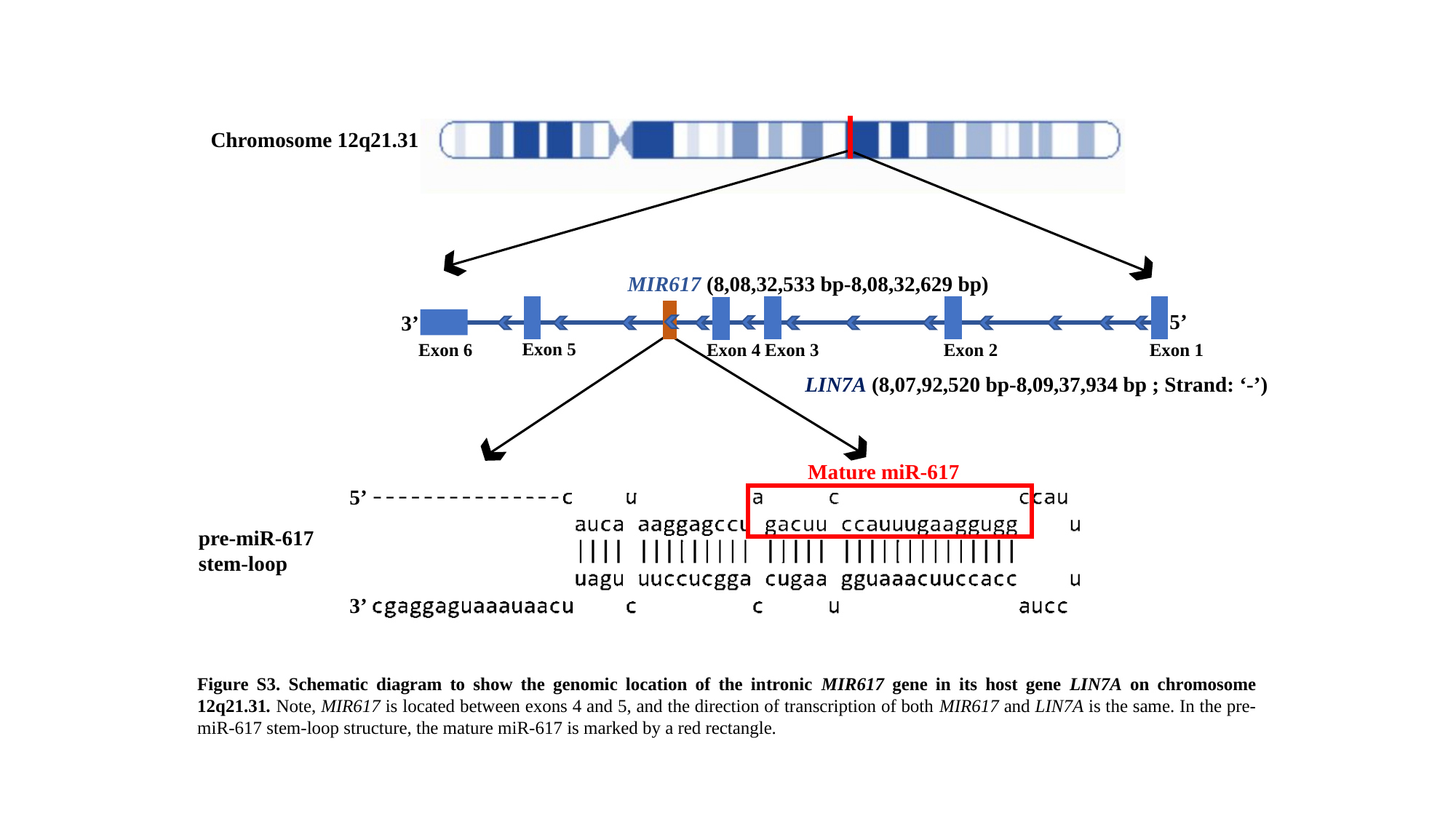

MIR617 (8,08,32,533 bp-8,08,32,629 bp)
5’
3’
Exon 5
Exon 6
Exon 3
Exon 4
Exon 2
LIN7A (8,07,92,520 bp-8,09,37,934 bp ; Strand: ‘-’)
5’
3’
Chromosome 12q21.31
Exon 1
pre-miR-617
stem-loop
Mature miR-617
Figure S3. Schematic diagram to show the genomic location of the intronic MIR617 gene in its host gene LIN7A on chromosome 12q21.31. Note, MIR617 is located between exons 4 and 5, and the direction of transcription of both MIR617 and LIN7A is the same. In the pre-miR-617 stem-loop structure, the mature miR-617 is marked by a red rectangle.

## Slide 5
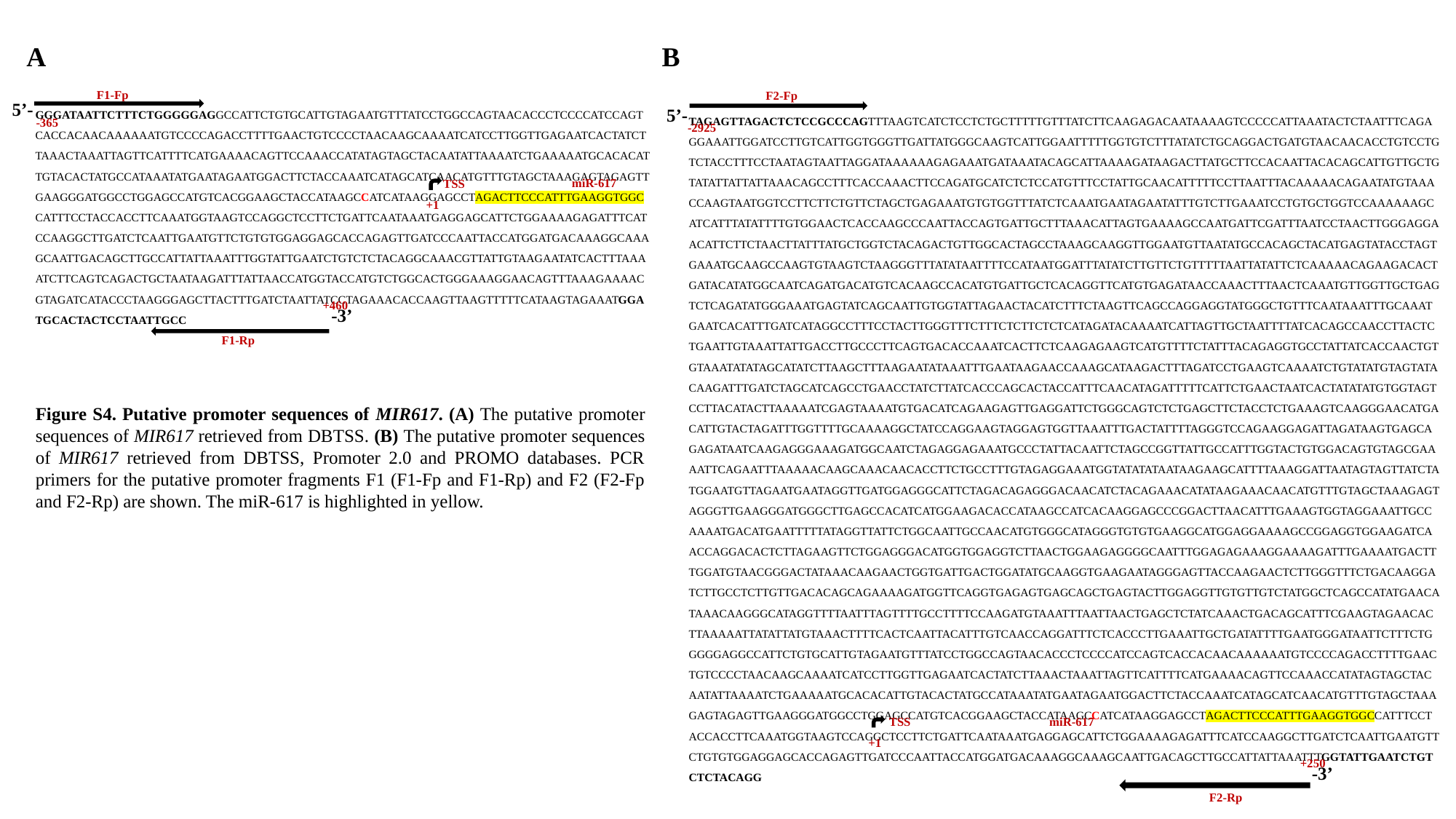

B
A
F1-Fp
5’-
GGGATAATTCTTTCTGGGGGAGGCCATTCTGTGCATTGTAGAATGTTTATCCTGGCCAGTAACACCCTCCCCATCCAGTCACCACAACAAAAAATGTCCCCAGACCTTTTGAACTGTCCCCTAACAAGCAAAATCATCCTTGGTTGAGAATCACTATCTTAAACTAAATTAGTTCATTTTCATGAAAACAGTTCCAAACCATATAGTAGCTACAATATTAAAATCTGAAAAATGCACACATTGTACACTATGCCATAAATATGAATAGAATGGACTTCTACCAAATCATAGCATCAACATGTTTGTAGCTAAAGAGTAGAGTTGAAGGGATGGCCTGGAGCCATGTCACGGAAGCTACCATAAGCCATCATAAGGAGCCTAGACTTCCCATTTGAAGGTGGCCATTTCCTACCACCTTCAAATGGTAAGTCCAGGCTCCTTCTGATTCAATAAATGAGGAGCATTCTGGAAAAGAGATTTCATCCAAGGCTTGATCTCAATTGAATGTTCTGTGTGGAGGAGCACCAGAGTTGATCCCAATTACCATGGATGACAAAGGCAAAGCAATTGACAGCTTGCCATTATTAAATTTGGTATTGAATCTGTCTCTACAGGCAAACGTTATTGTAAGAATATCACTTTAAAATCTTCAGTCAGACTGCTAATAAGATTTATTAACCATGGTACCATGTCTGGCACTGGGAAAGGAACAGTTTAAAGAAAACGTAGATCATACCCTAAGGGAGCTTACTTTGATCTAATTATCCTAGAAACACCAAGTTAAGTTTTTCATAAGTAGAAATGGATGCACTACTCCTAATTGCC
-365
miR-617
TSS
+1
+460
-3’
F1-Rp
F2-Fp
5’-
TAGAGTTAGACTCTCCGCCCAGTTTAAGTCATCTCCTCTGCTTTTTGTTTATCTTCAAGAGACAATAAAAGTCCCCCATTAAATACTCTAATTTCAGAGGAAATTGGATCCTTGTCATTGGTGGGTTGATTATGGGCAAGTCATTGGAATTTTTGGTGTCTTTATATCTGCAGGACTGATGTAACAACACCTGTCCTGTCTACCTTTCCTAATAGTAATTAGGATAAAAAAGAGAAATGATAAATACAGCATTAAAAGATAAGACTTATGCTTCCACAATTACACAGCATTGTTGCTGTATATTATTATTAAACAGCCTTTCACCAAACTTCCAGATGCATCTCTCCATGTTTCCTATTGCAACATTTTTCCTTAATTTACAAAAACAGAATATGTAAACCAAGTAATGGTCCTTCTTCTGTTCTAGCTGAGAAATGTGTGGTTTATCTCAAATGAATAGAATATTTGTCTTGAAATCCTGTGCTGGTCCAAAAAAGCATCATTTATATTTTGTGGAACTCACCAAGCCCAATTACCAGTGATTGCTTTAAACATTAGTGAAAAGCCAATGATTCGATTTAATCCTAACTTGGGAGGAACATTCTTCTAACTTATTTATGCTGGTCTACAGACTGTTGGCACTAGCCTAAAGCAAGGTTGGAATGTTAATATGCCACAGCTACATGAGTATACCTAGTGAAATGCAAGCCAAGTGTAAGTCTAAGGGTTTATATAATTTTCCATAATGGATTTATATCTTGTTCTGTTTTTAATTATATTCTCAAAAACAGAAGACACTGATACATATGGCAATCAGATGACATGTCACAAGCCACATGTGATTGCTCACAGGTTCATGTGAGATAACCAAACTTTAACTCAAATGTTGGTTGCTGAGTCTCAGATATGGGAAATGAGTATCAGCAATTGTGGTATTAGAACTACATCTTTCTAAGTTCAGCCAGGAGGTATGGGCTGTTTCAATAAATTTGCAAATGAATCACATTTGATCATAGGCCTTTCCTACTTGGGTTTCTTTCTCTTCTCTCATAGATACAAAATCATTAGTTGCTAATTTTATCACAGCCAACCTTACTCTGAATTGTAAATTATTGACCTTGCCCTTCAGTGACACCAAATCACTTCTCAAGAGAAGTCATGTTTTCTATTTACAGAGGTGCCTATTATCACCAACTGTGTAAATATATAGCATATCTTAAGCTTTAAGAATATAAATTTGAATAAGAACCAAAGCATAAGACTTTAGATCCTGAAGTCAAAATCTGTATATGTAGTATACAAGATTTGATCTAGCATCAGCCTGAACCTATCTTATCACCCAGCACTACCATTTCAACATAGATTTTTCATTCTGAACTAATCACTATATATGTGGTAGTCCTTACATACTTAAAAATCGAGTAAAATGTGACATCAGAAGAGTTGAGGATTCTGGGCAGTCTCTGAGCTTCTACCTCTGAAAGTCAAGGGAACATGACATTGTACTAGATTTGGTTTTGCAAAAGGCTATCCAGGAAGTAGGAGTGGTTAAATTTGACTATTTTAGGGTCCAGAAGGAGATTAGATAAGTGAGCAGAGATAATCAAGAGGGAAAGATGGCAATCTAGAGGAGAAATGCCCTATTACAATTCTAGCCGGTTATTGCCATTTGGTACTGTGGACAGTGTAGCGAAAATTCAGAATTTAAAAACAAGCAAACAACACCTTCTGCCTTTGTAGAGGAAATGGTATATATAATAAGAAGCATTTTAAAGGATTAATAGTAGTTATCTATGGAATGTTAGAATGAATAGGTTGATGGAGGGCATTCTAGACAGAGGGACAACATCTACAGAAACATATAAGAAACAACATGTTTGTAGCTAAAGAGTAGGGTTGAAGGGATGGGCTTGAGCCACATCATGGAAGACACCATAAGCCATCACAAGGAGCCCGGACTTAACATTTGAAAGTGGTAGGAAATTGCCAAAATGACATGAATTTTTATAGGTTATTCTGGCAATTGCCAACATGTGGGCATAGGGTGTGTGAAGGCATGGAGGAAAAGCCGGAGGTGGAAGATCAACCAGGACACTCTTAGAAGTTCTGGAGGGACATGGTGGAGGTCTTAACTGGAAGAGGGGCAATTTGGAGAGAAAGGAAAAGATTTGAAAATGACTTTGGATGTAACGGGACTATAAACAAGAACTGGTGATTGACTGGATATGCAAGGTGAAGAATAGGGAGTTACCAAGAACTCTTGGGTTTCTGACAAGGATCTTGCCTCTTGTTGACACAGCAGAAAAGATGGTTCAGGTGAGAGTGAGCAGCTGAGTACTTGGAGGTTGTGTTGTCTATGGCTCAGCCATATGAACATAAACAAGGGCATAGGTTTTAATTTAGTTTTGCCTTTTCCAAGATGTAAATTTAATTAACTGAGCTCTATCAAACTGACAGCATTTCGAAGTAGAACACTTAAAAATTATATTATGTAAACTTTTCACTCAATTACATTTGTCAACCAGGATTTCTCACCCTTGAAATTGCTGATATTTTGAATGGGATAATTCTTTCTGGGGGAGGCCATTCTGTGCATTGTAGAATGTTTATCCTGGCCAGTAACACCCTCCCCATCCAGTCACCACAACAAAAAATGTCCCCAGACCTTTTGAACTGTCCCCTAACAAGCAAAATCATCCTTGGTTGAGAATCACTATCTTAAACTAAATTAGTTCATTTTCATGAAAACAGTTCCAAACCATATAGTAGCTACAATATTAAAATCTGAAAAATGCACACATTGTACACTATGCCATAAATATGAATAGAATGGACTTCTACCAAATCATAGCATCAACATGTTTGTAGCTAAAGAGTAGAGTTGAAGGGATGGCCTGGAGCCATGTCACGGAAGCTACCATAAGCCATCATAAGGAGCCTAGACTTCCCATTTGAAGGTGGCCATTTCCTACCACCTTCAAATGGTAAGTCCAGGCTCCTTCTGATTCAATAAATGAGGAGCATTCTGGAAAAGAGATTTCATCCAAGGCTTGATCTCAATTGAATGTTCTGTGTGGAGGAGCACCAGAGTTGATCCCAATTACCATGGATGACAAAGGCAAAGCAATTGACAGCTTGCCATTATTAAATTTGGTATTGAATCTGTCTCTACAGG
-2925
TSS
miR-617
+1
+250
-3’
F2-Rp
Figure S4. Putative promoter sequences of MIR617. (A) The putative promoter sequences of MIR617 retrieved from DBTSS. (B) The putative promoter sequences of MIR617 retrieved from DBTSS, Promoter 2.0 and PROMO databases. PCR primers for the putative promoter fragments F1 (F1-Fp and F1-Rp) and F2 (F2-Fp and F2-Rp) are shown. The miR-617 is highlighted in yellow.

## Slide 6
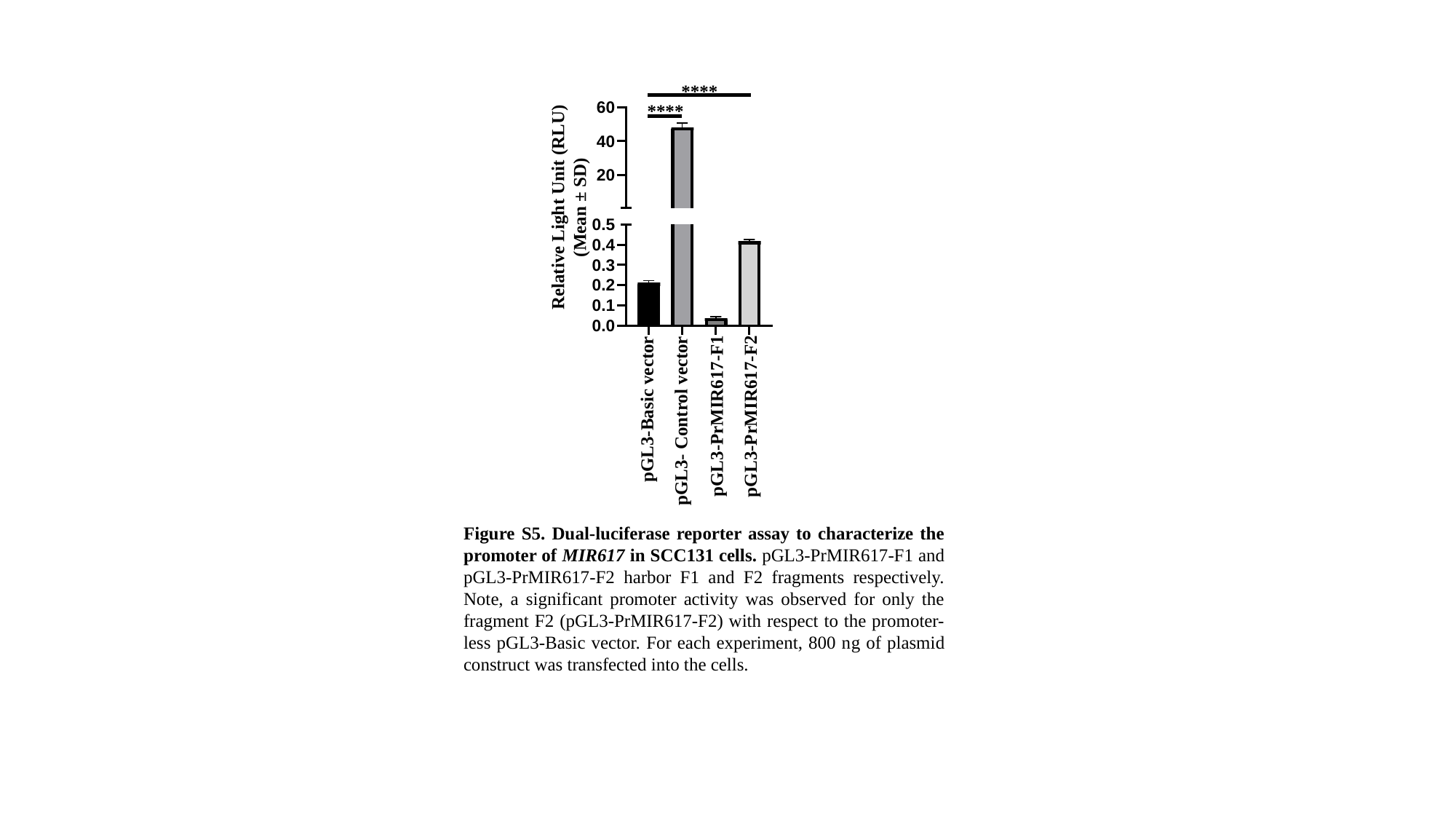

****
****
pGL3-PrMIR617-F2
pGL3-Basic vector
pGL3- Control vector
Relative Light Unit (RLU)
(Mean ± SD)
pGL3-PrMIR617-F1
Figure S5. Dual-luciferase reporter assay to characterize the promoter of MIR617 in SCC131 cells. pGL3-PrMIR617-F1 and pGL3-PrMIR617-F2 harbor F1 and F2 fragments respectively. Note, a significant promoter activity was observed for only the fragment F2 (pGL3-PrMIR617-F2) with respect to the promoter-less pGL3-Basic vector. For each experiment, 800 ng of plasmid construct was transfected into the cells.

## Slide 7
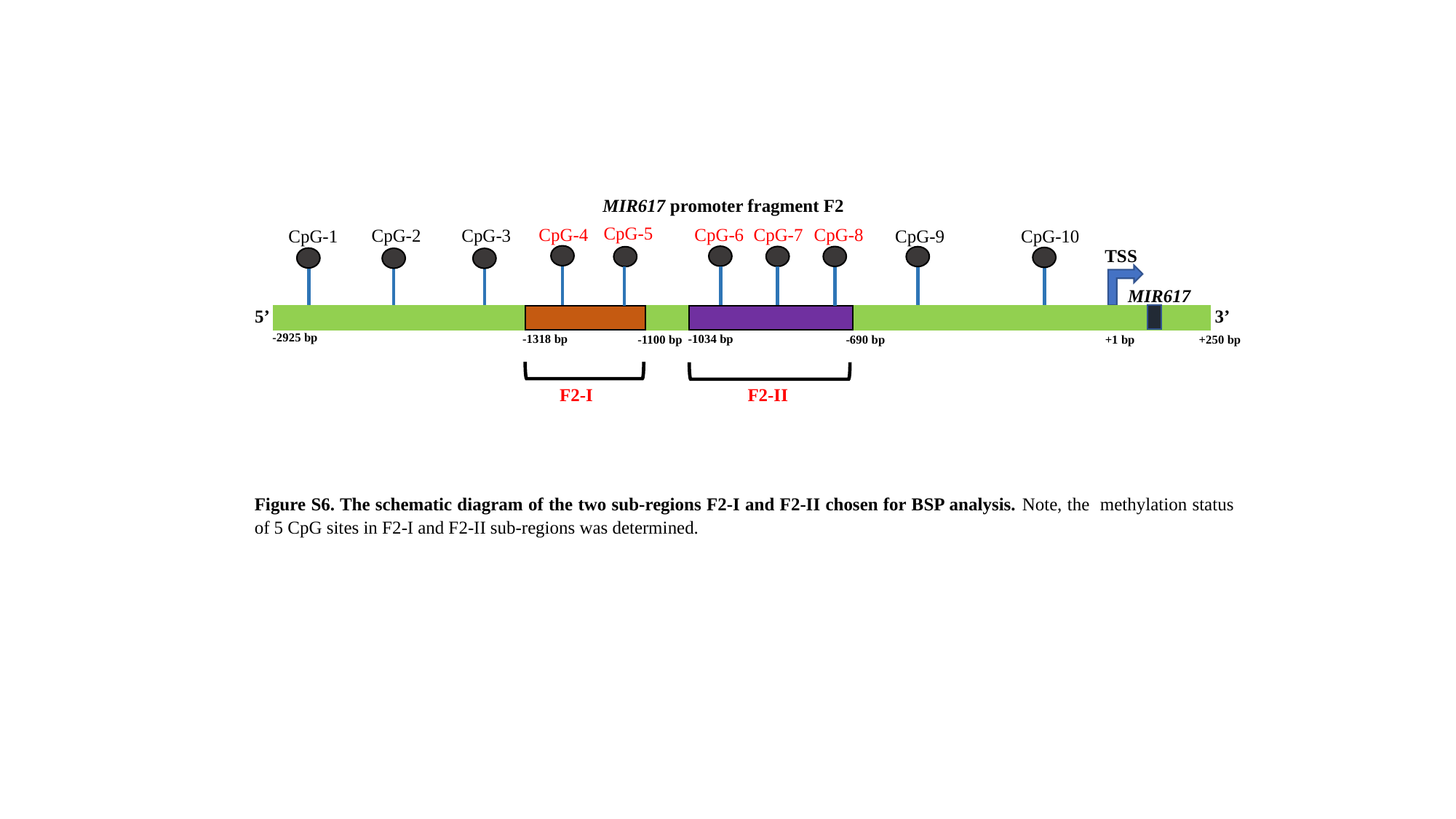

MIR617 promoter fragment F2
TSS
MIR617
5’
4th intron of LIN7A
-2925 bp
+250 bp
CpG-5
CpG-6
CpG-4
CpG-7
CpG-8
CpG-2
CpG-3
CpG-1
CpG-10
CpG-9
3’
+1 bp
-1034 bp
-1318 bp
-1100 bp
-690 bp
F2-I
F2-II
Figure S6. The schematic diagram of the two sub-regions F2-I and F2-II chosen for BSP analysis. Note, the methylation status of 5 CpG sites in F2-I and F2-II sub-regions was determined.

## Slide 8
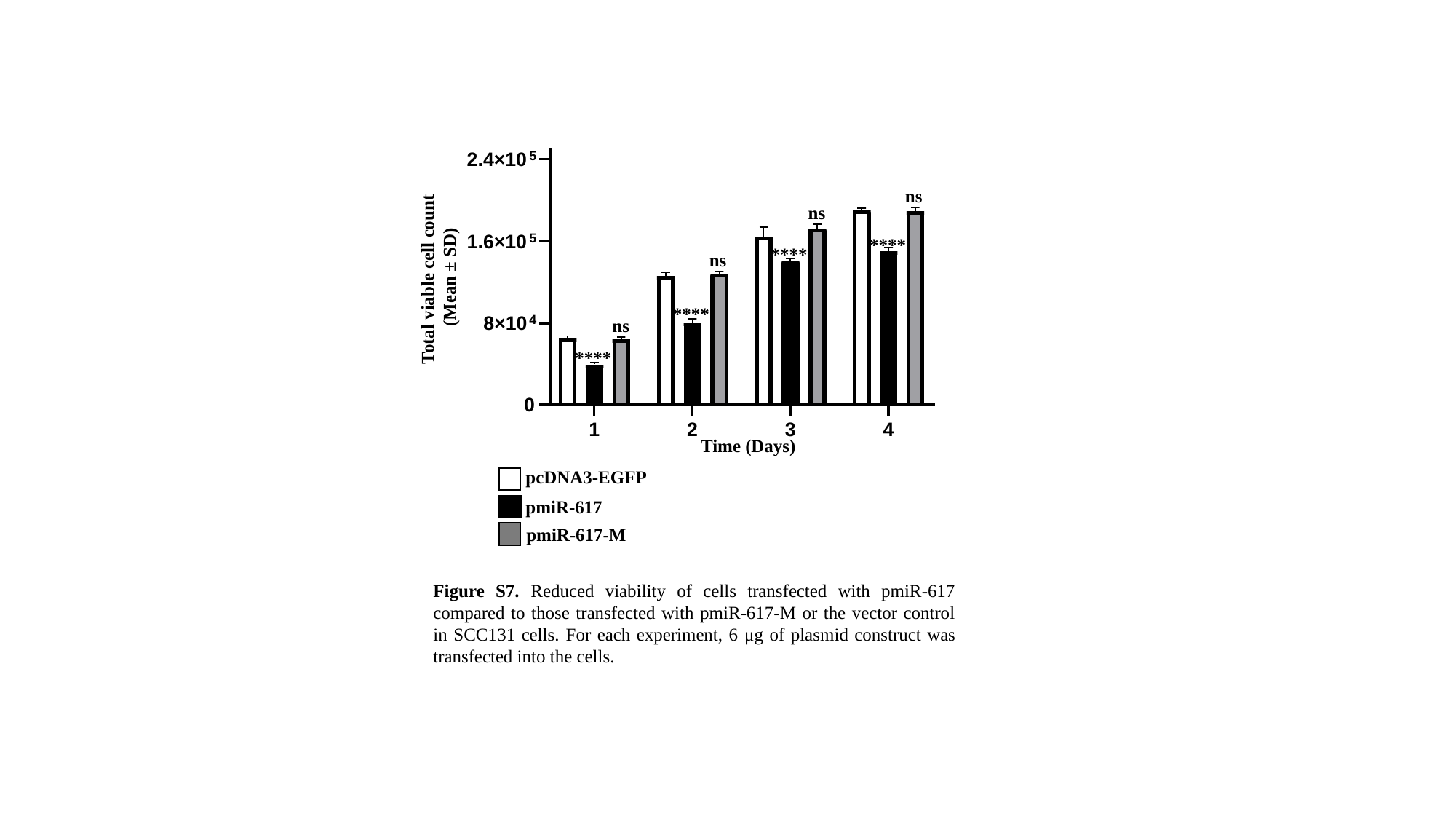

Total viable cell count
(Mean ± SD)
ns
ns
****
****
ns
****
ns
****
Time (Days)
pcDNA3-EGFP
pmiR-617
pmiR-617-M
Figure S7. Reduced viability of cells transfected with pmiR-617 compared to those transfected with pmiR-617-M or the vector control in SCC131 cells. For each experiment, 6 μg of plasmid construct was transfected into the cells.

## Slide 9
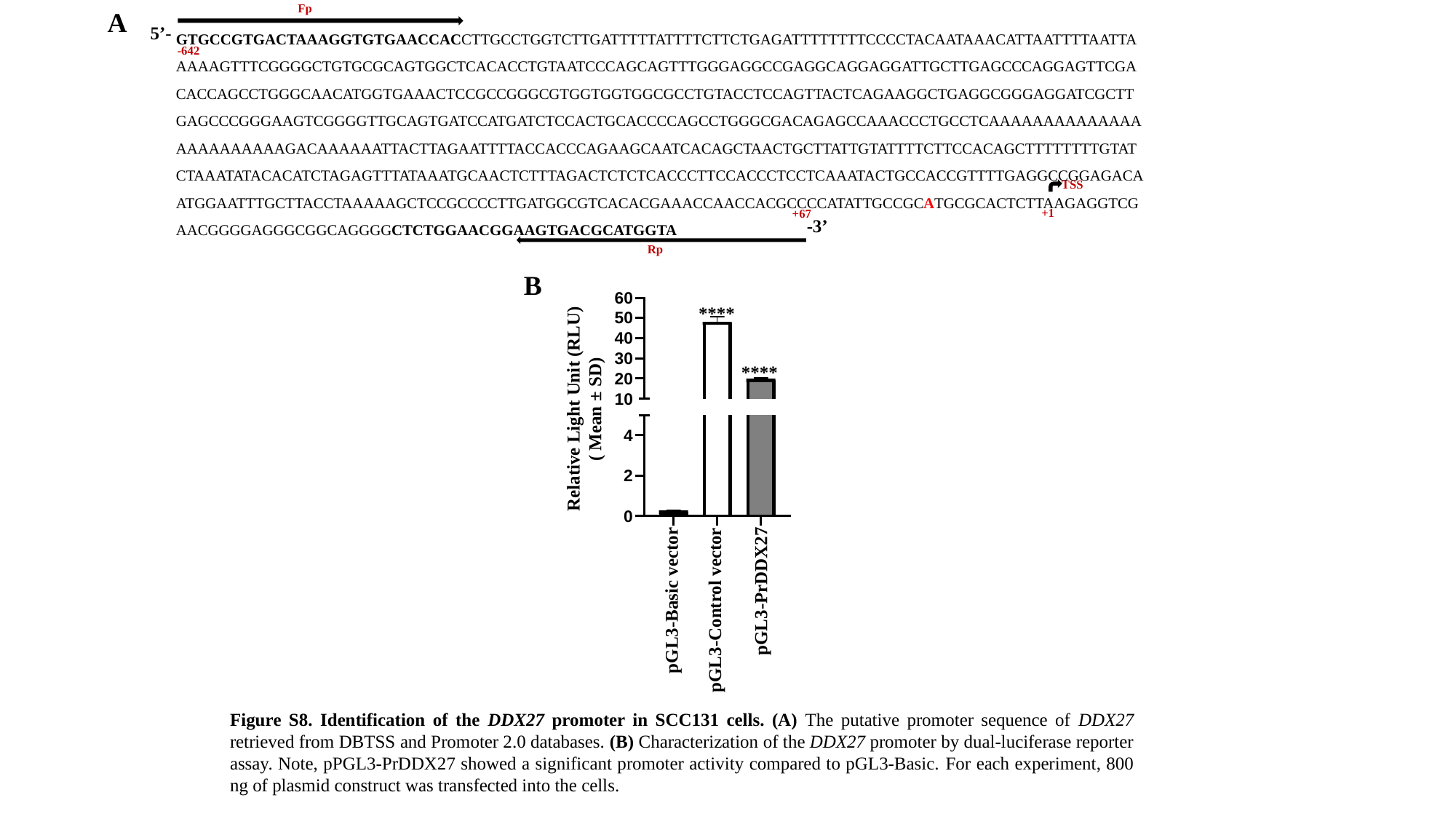

Fp
GTGCCGTGACTAAAGGTGTGAACCACCTTGCCTGGTCTTGATTTTTATTTTCTTCTGAGATTTTTTTTCCCCTACAATAAACATTAATTTTAATTAAAAAGTTTCGGGGCTGTGCGCAGTGGCTCACACCTGTAATCCCAGCAGTTTGGGAGGCCGAGGCAGGAGGATTGCTTGAGCCCAGGAGTTCGACACCAGCCTGGGCAACATGGTGAAACTCCGCCGGGCGTGGTGGTGGCGCCTGTACCTCCAGTTACTCAGAAGGCTGAGGCGGGAGGATCGCTTGAGCCCGGGAAGTCGGGGTTGCAGTGATCCATGATCTCCACTGCACCCCAGCCTGGGCGACAGAGCCAAACCCTGCCTCAAAAAAAAAAAAAAAAAAAAAAAAGACAAAAAATTACTTAGAATTTTACCACCCAGAAGCAATCACAGCTAACTGCTTATTGTATTTTCTTCCACAGCTTTTTTTTGTATCTAAATATACACATCTAGAGTTTATAAATGCAACTCTTTAGACTCTCTCACCCTTCCACCCTCCTCAAATACTGCCACCGTTTTGAGGCCGGAGACAATGGAATTTGCTTACCTAAAAAGCTCCGCCCCTTGATGGCGTCACACGAAACCAACCACGCCCCATATTGCCGCATGCGCACTCTTAAGAGGTCGAACGGGGAGGGCGGCAGGGGCTCTGGAACGGAAGTGACGCATGGTA
5’-
-642
TSS
+1
+67
-3’
Rp
A
B
****
pGL3-PrDDX27
pGL3-Basic vector
pGL3-Control vector
Relative Light Unit (RLU)
 ( Mean ± SD)
****
Figure S8. Identification of the DDX27 promoter in SCC131 cells. (A) The putative promoter sequence of DDX27 retrieved from DBTSS and Promoter 2.0 databases. (B) Characterization of the DDX27 promoter by dual-luciferase reporter assay. Note, pPGL3-PrDDX27 showed a significant promoter activity compared to pGL3-Basic. For each experiment, 800 ng of plasmid construct was transfected into the cells.

## Slide 10
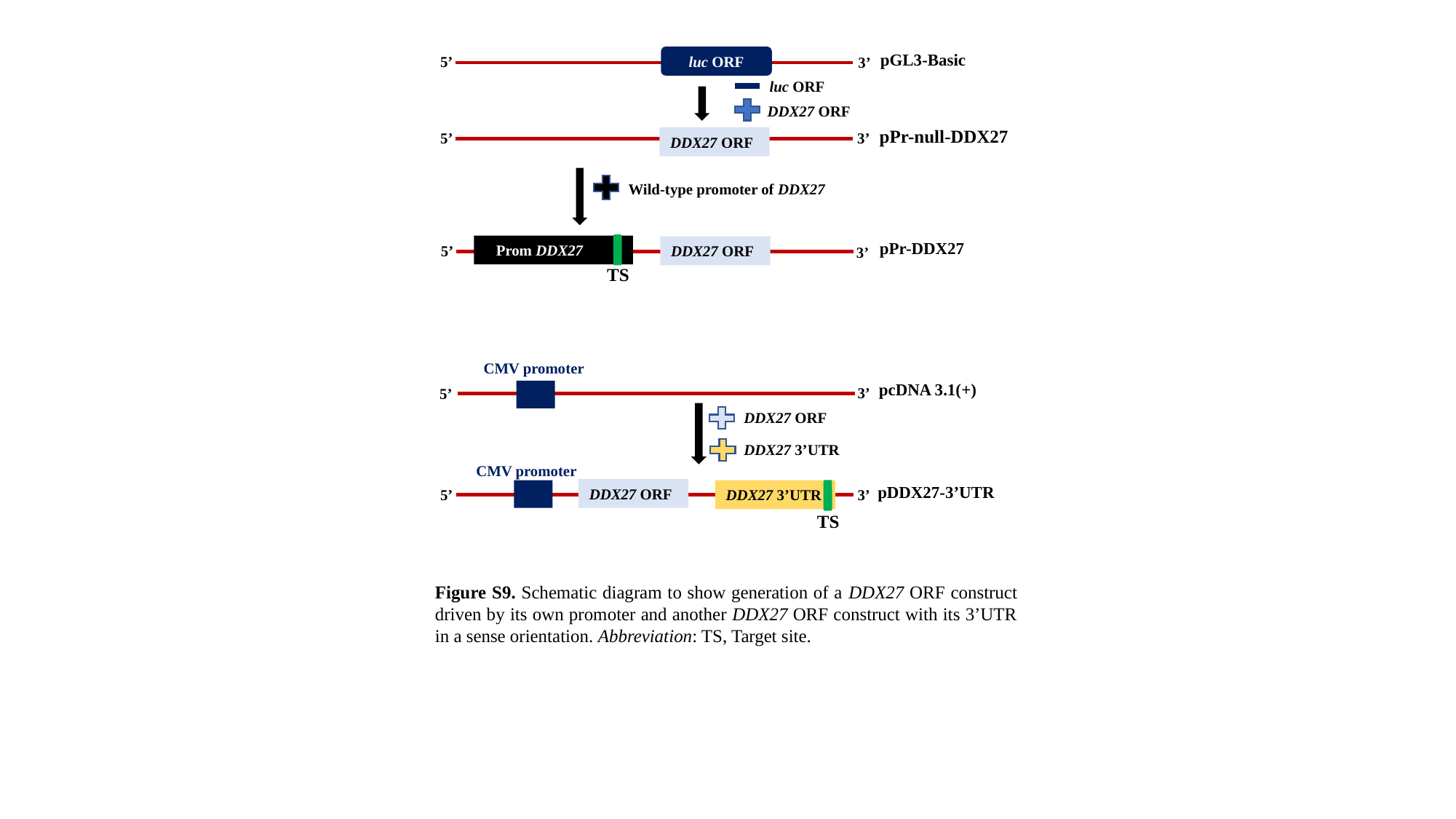

pGL3-Basic
luc ORF
 5’
3’
Wild-type promoter of DDX27
pPr-DDX27
 Prom DDX27
5’
DDX27 ORF
3’
CMV promoter
pcDNA 3.1(+)
3’
5’
DDX27 ORF
DDX27 3’UTR
pDDX27-3’UTR
DDX27 ORF
3’
5’
DDX27 3’UTR
luc ORF
DDX27 ORF
pPr-null-DDX27
 5’
3’
DDX27 ORF
TS
CMV promoter
TS
Figure S9. Schematic diagram to show generation of a DDX27 ORF construct driven by its own promoter and another DDX27 ORF construct with its 3’UTR in a sense orientation. Abbreviation: TS, Target site.

## Slide 11
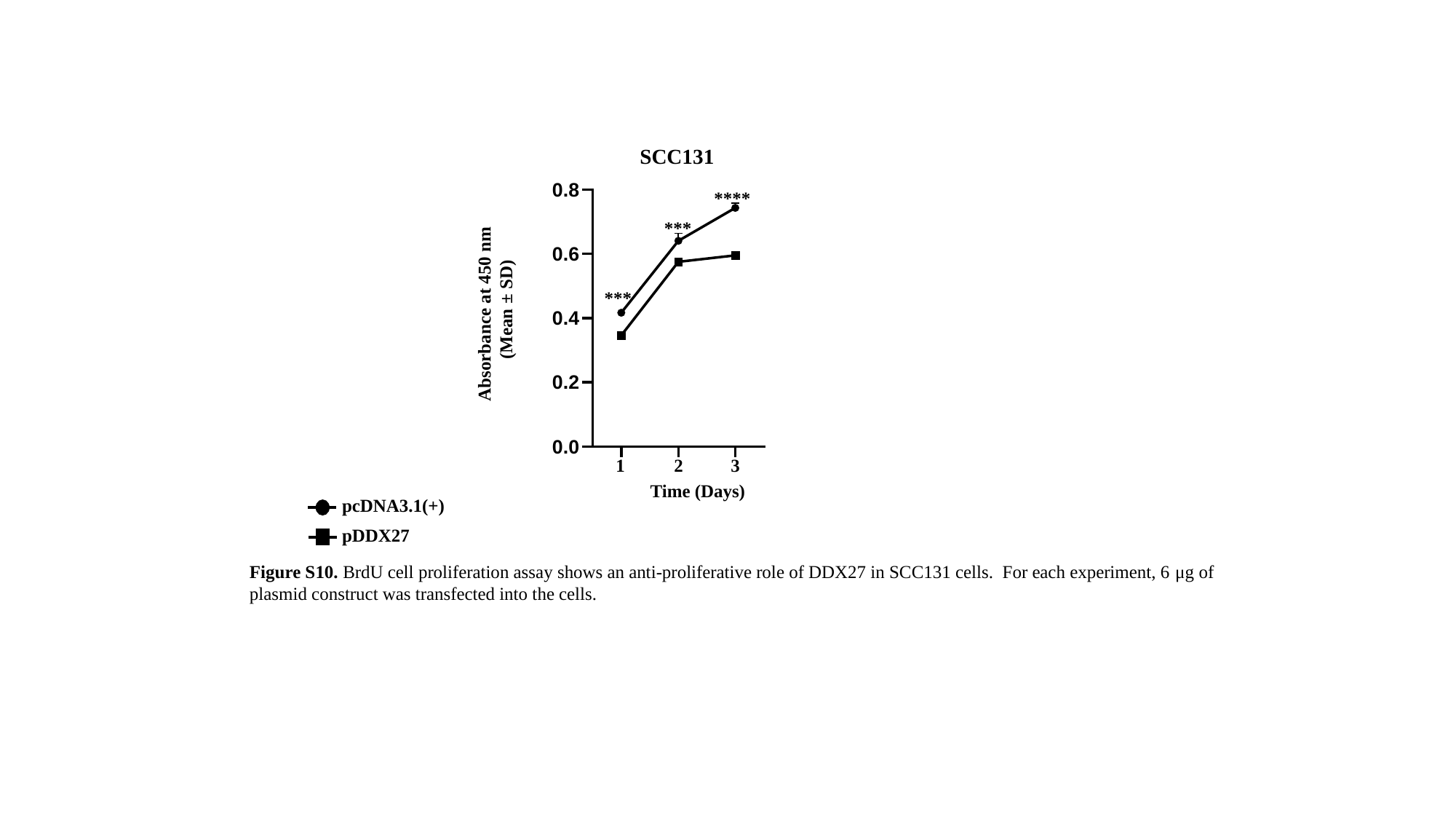

SCC131
Absorbance at 450 nm
 (Mean ± SD)
1
3
2
****
***
***
Time (Days)
pcDNA3.1(+)
pDDX27
Figure S10. BrdU cell proliferation assay shows an anti-proliferative role of DDX27 in SCC131 cells. For each experiment, 6 μg of plasmid construct was transfected into the cells.

## Slide 12
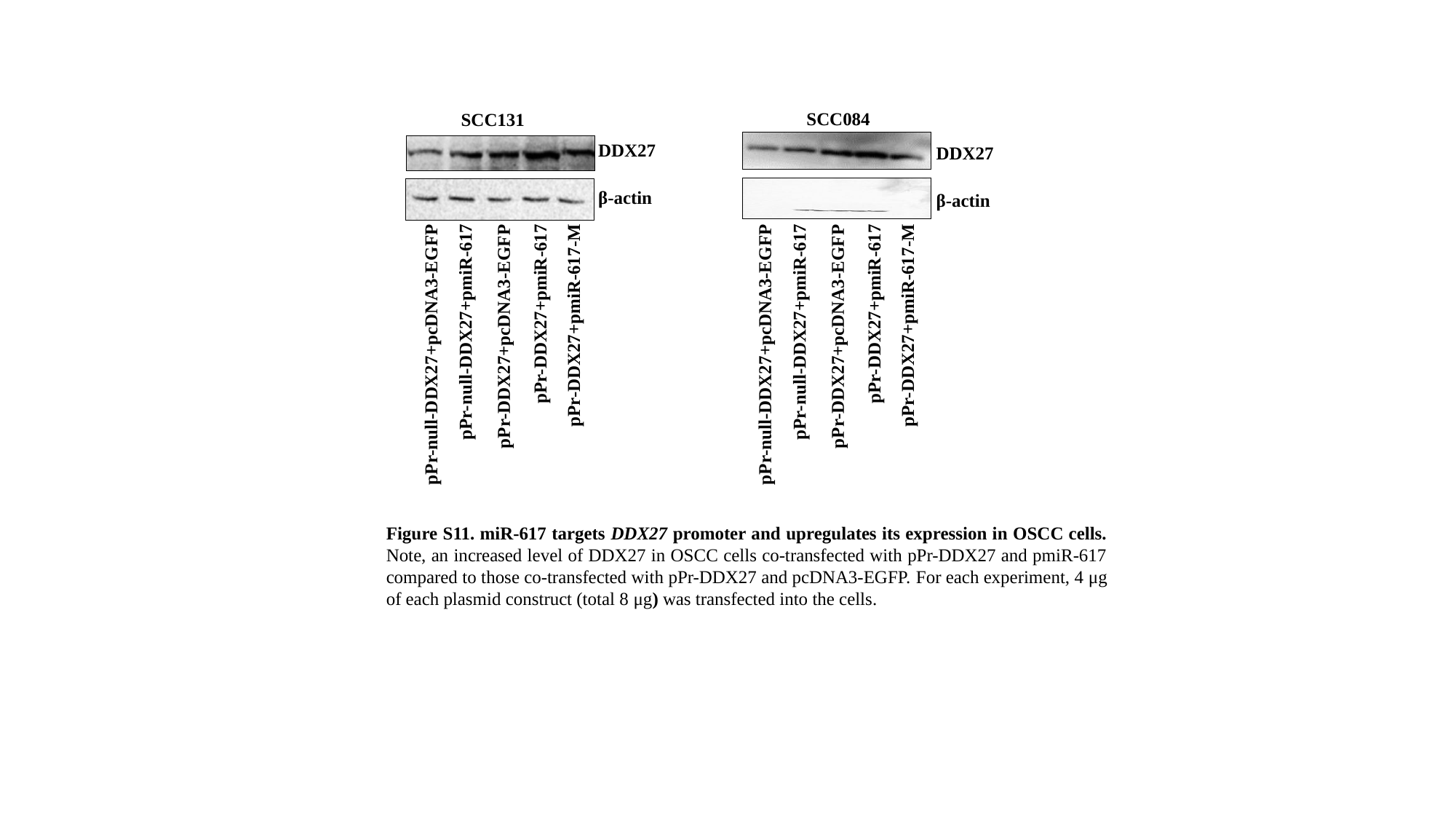

DDX27
β-actin
pPr-DDX27+pmiR-617
pPr-DDX27+pmiR-617-M
pPr-DDX27+pcDNA3-EGFP
pPr-null-DDX27+pmiR-617
pPr-null-DDX27+pcDNA3-EGFP
DDX27
β-actin
pPr-DDX27+pmiR-617
pPr-DDX27+pmiR-617-M
pPr-DDX27+pcDNA3-EGFP
pPr-null-DDX27+pmiR-617
pPr-null-DDX27+pcDNA3-EGFP
SCC084
SCC131
Figure S11. miR-617 targets DDX27 promoter and upregulates its expression in OSCC cells. Note, an increased level of DDX27 in OSCC cells co-transfected with pPr-DDX27 and pmiR-617 compared to those co-transfected with pPr-DDX27 and pcDNA3-EGFP. For each experiment, 4 μg of each plasmid construct (total 8 μg) was transfected into the cells.

## Slide 13
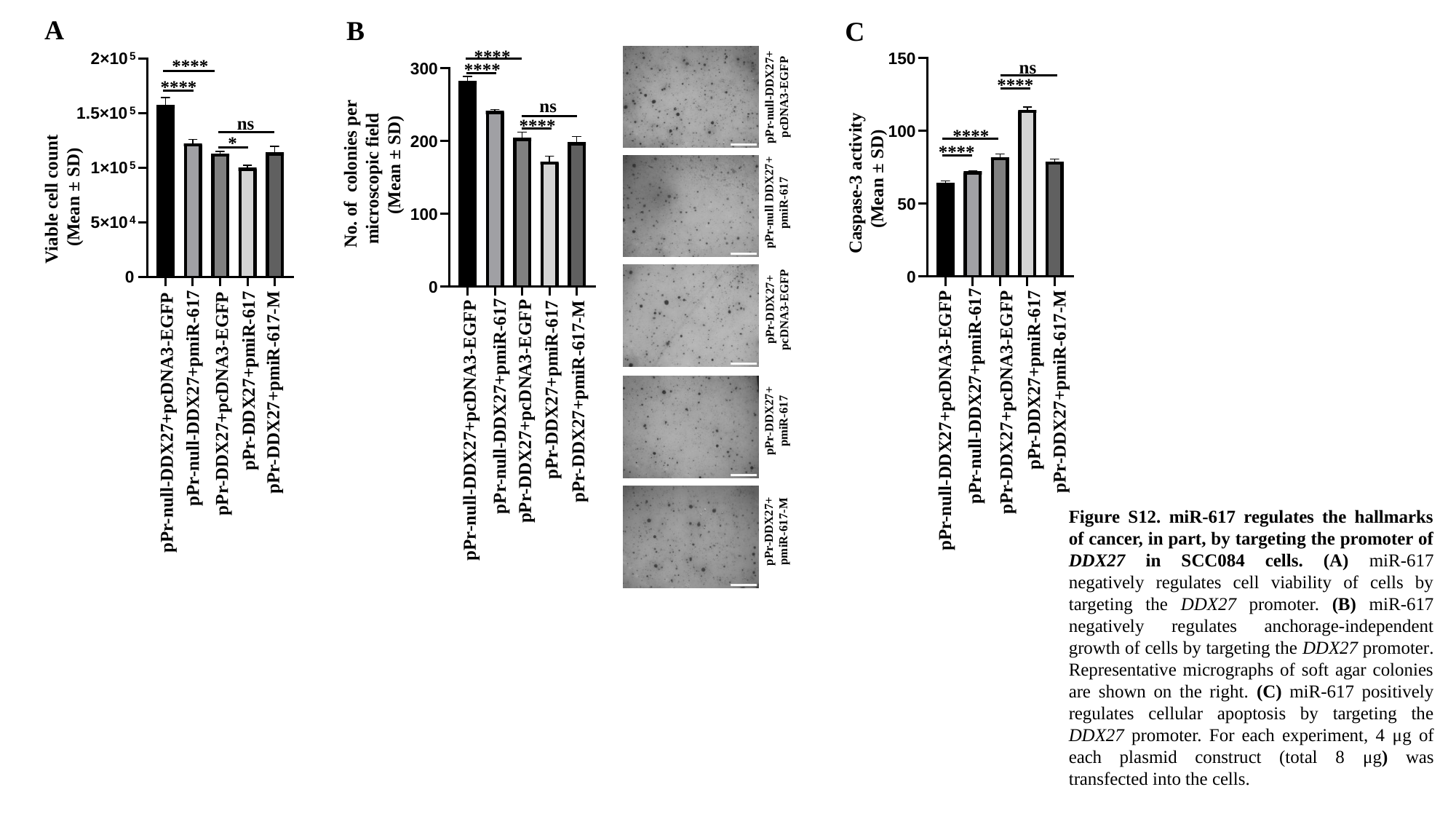

A
ns
 ****
****
****
Caspase-3 activity
(Mean ± SD)
pPr-DDX27+pmiR-617
pPr-DDX27+pmiR-617-M
pPr-DDX27+pcDNA3-EGFP
pPr-null-DDX27+pmiR-617
pPr-null-DDX27+pcDNA3-EGFP
C
****
****
ns
 *
Viable cell count
 (Mean ± SD)
pPr-DDX27+pmiR-617
pPr-DDX27+pmiR-617-M
pPr-DDX27+pcDNA3-EGFP
pPr-null-DDX27+pmiR-617
pPr-null-DDX27+pcDNA3-EGFP
B
****
****
ns
****
 No. of colonies per
 microscopic field
 (Mean ± SD)
pPr-DDX27+pmiR-617
pPr-DDX27+pmiR-617-M
pPr-DDX27+pcDNA3-EGFP
pPr-null-DDX27+pmiR-617
pPr-null-DDX27+pcDNA3-EGFP
pPr-null-DDX27+
pcDNA3-EGFP
pPr-null DDX27+
pmiR-617
pPr-DDX27+
pcDNA3-EGFP
pPr-DDX27+
pmiR-617
pPr-DDX27+
pmiR-617-M
Figure S12. miR-617 regulates the hallmarks of cancer, in part, by targeting the promoter of DDX27 in SCC084 cells. (A) miR-617 negatively regulates cell viability of cells by targeting the DDX27 promoter. (B) miR-617 negatively regulates anchorage-independent growth of cells by targeting the DDX27 promoter. Representative micrographs of soft agar colonies are shown on the right. (C) miR-617 positively regulates cellular apoptosis by targeting the DDX27 promoter. For each experiment, 4 μg of each plasmid construct (total 8 μg) was transfected into the cells.

## Slide 14
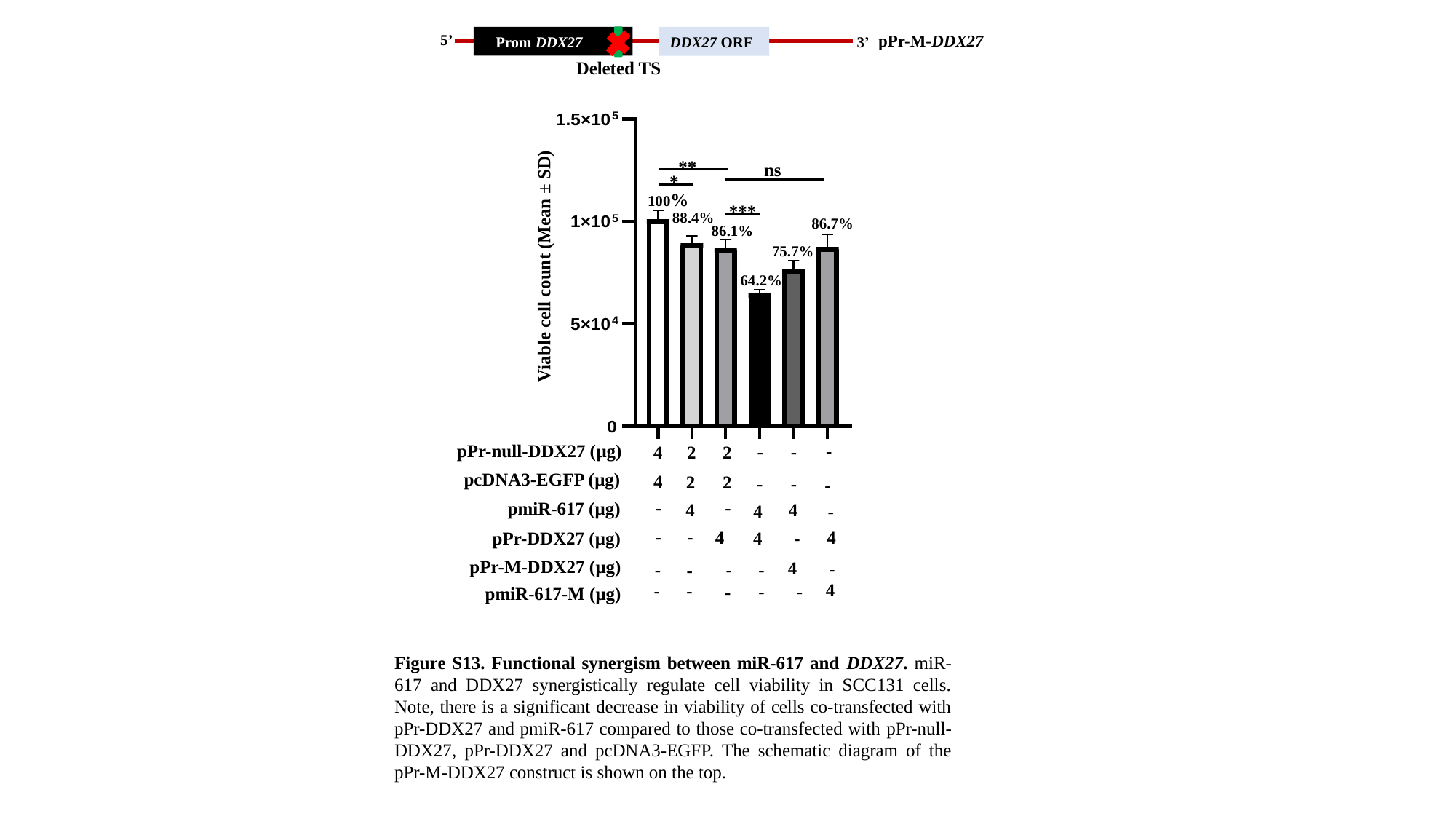

pPr-M-DDX27
5’
 Prom DDX27
DDX27 ORF
3’
Deleted TS
**
*
100%
***
Viable cell count (Mean ± SD)
88.4%
86.1%
75.7%
64.2%
pPr-null-DDX27 (μg)
-
-
4
2
2
pcDNA3-EGFP (μg)
4
2
2
-
-
-
-
pmiR-617 (μg)
4
4
4
-
-
4
4
pPr-DDX27 (μg)
-
pPr-M-DDX27 (μg)
4
-
-
-
-
ns
86.7%
-
-
-
4
-
4
-
-
-
-
-
pmiR-617-M (μg)
Figure S13. Functional synergism between miR-617 and DDX27. miR-617 and DDX27 synergistically regulate cell viability in SCC131 cells. Note, there is a significant decrease in viability of cells co-transfected with pPr-DDX27 and pmiR-617 compared to those co-transfected with pPr-null-DDX27, pPr-DDX27 and pcDNA3-EGFP. The schematic diagram of the pPr-M-DDX27 construct is shown on the top.

## Slide 15
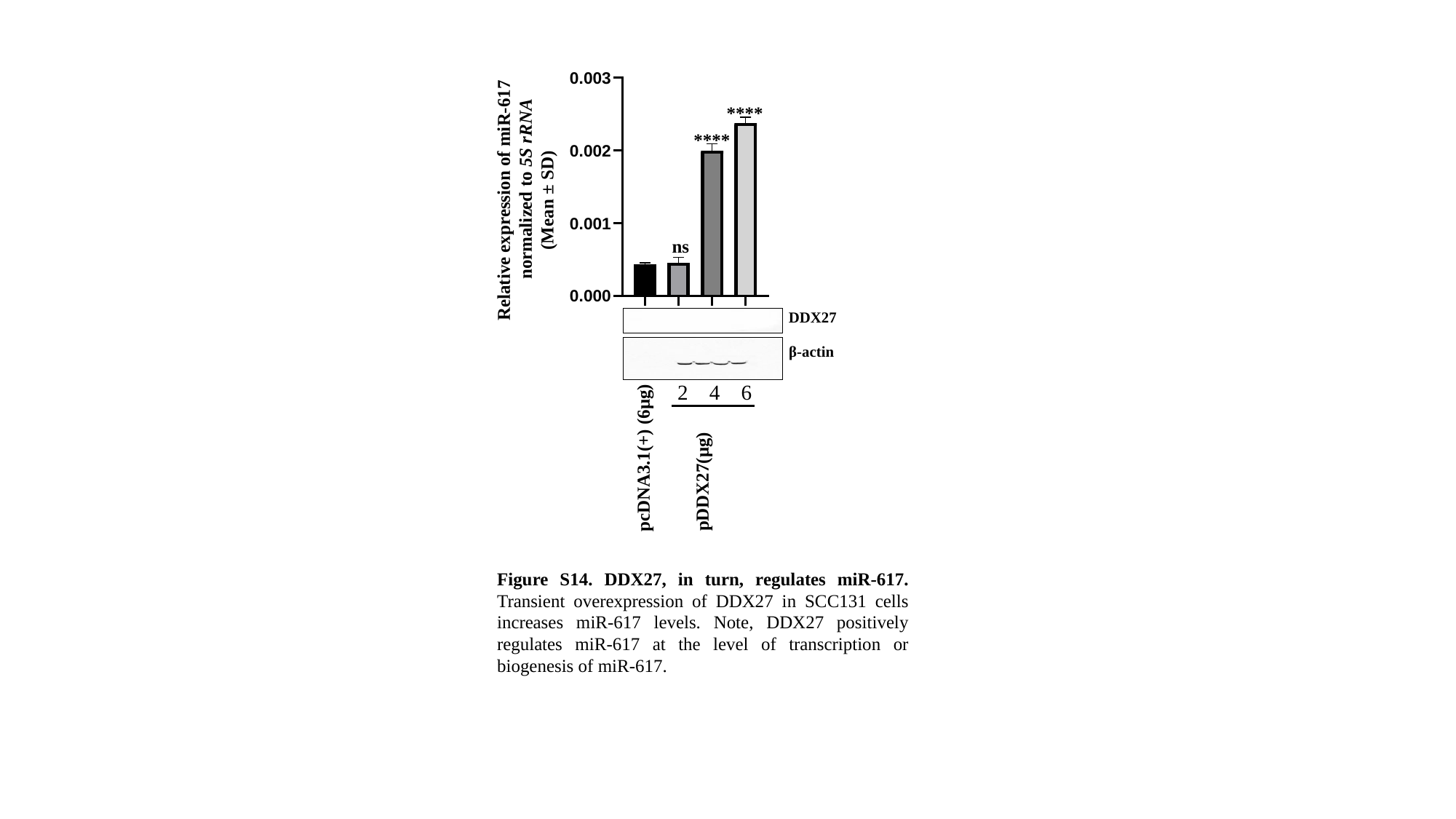

****
****
Relative expression of miR-617
 normalized to 5S rRNA
(Mean ± SD)
ns
DDX27
β-actin
 2 4 6
pDDX27(μg)
pcDNA3.1(+) (6μg)
Figure S14. DDX27, in turn, regulates miR-617. Transient overexpression of DDX27 in SCC131 cells increases miR-617 levels. Note, DDX27 positively regulates miR-617 at the level of transcription or biogenesis of miR-617.

## Slide 16
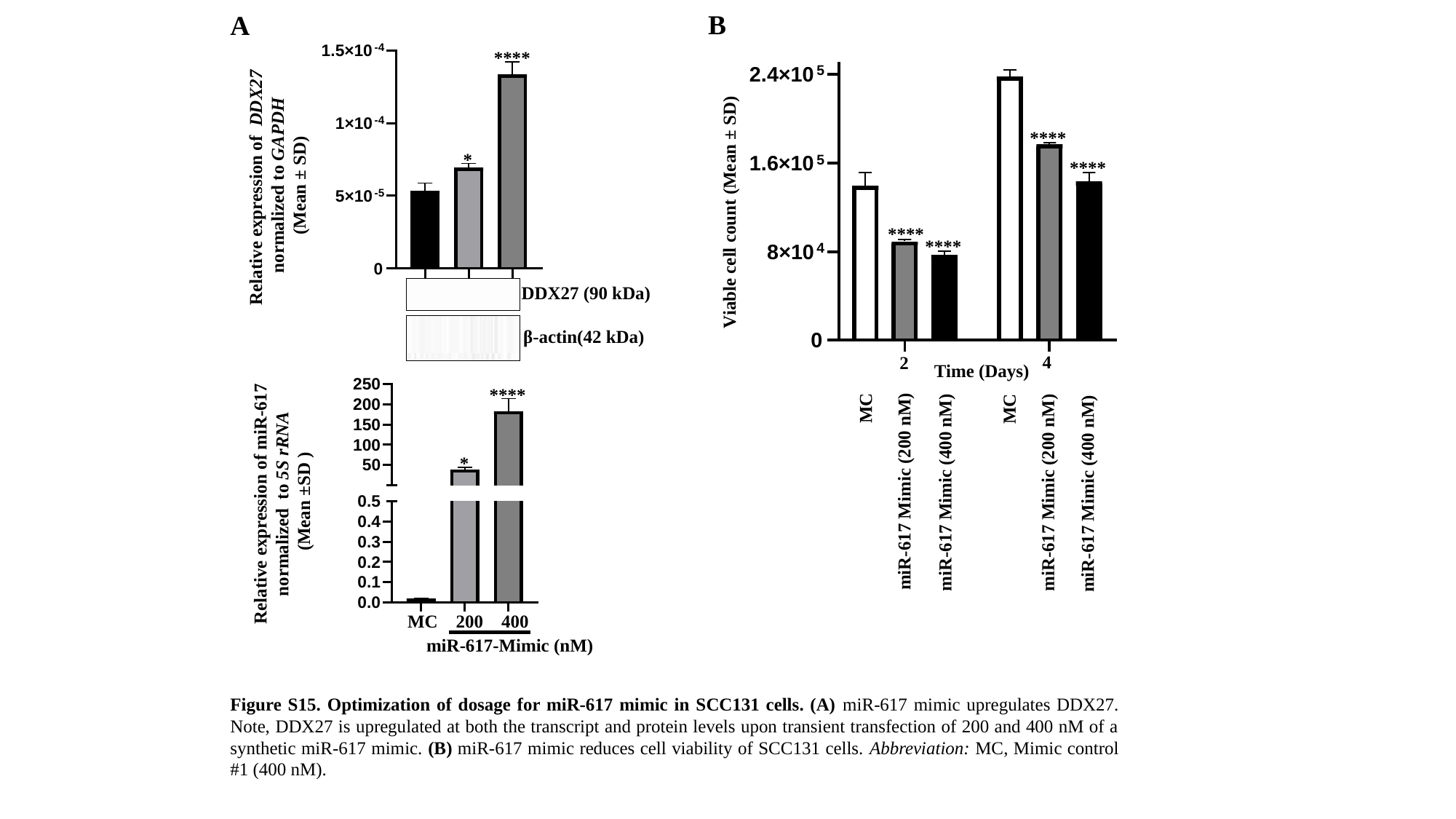

Viable cell count (Mean ± SD)
****
****
****
****
4
2
Time (Days)
MC
MC
miR-617 Mimic (200 nM)
miR-617 Mimic (400 nM)
miR-617 Mimic (200 nM)
miR-617 Mimic (400 nM)
B
A
*
Relative expression of DDX27
normalized to GAPDH
(Mean ± SD)
DDX27 (90 kDa)
β-actin(42 kDa)
****
*
MC 200 400
 miR-617-Mimic (nM)
Relative expression of miR-617 normalized to 5S rRNA
 (Mean ±SD )
****
Figure S15. Optimization of dosage for miR-617 mimic in SCC131 cells. (A) miR-617 mimic upregulates DDX27. Note, DDX27 is upregulated at both the transcript and protein levels upon transient transfection of 200 and 400 nM of a synthetic miR-617 mimic. (B) miR-617 mimic reduces cell viability of SCC131 cells. Abbreviation: MC, Mimic control #1 (400 nM).

## Slide 17
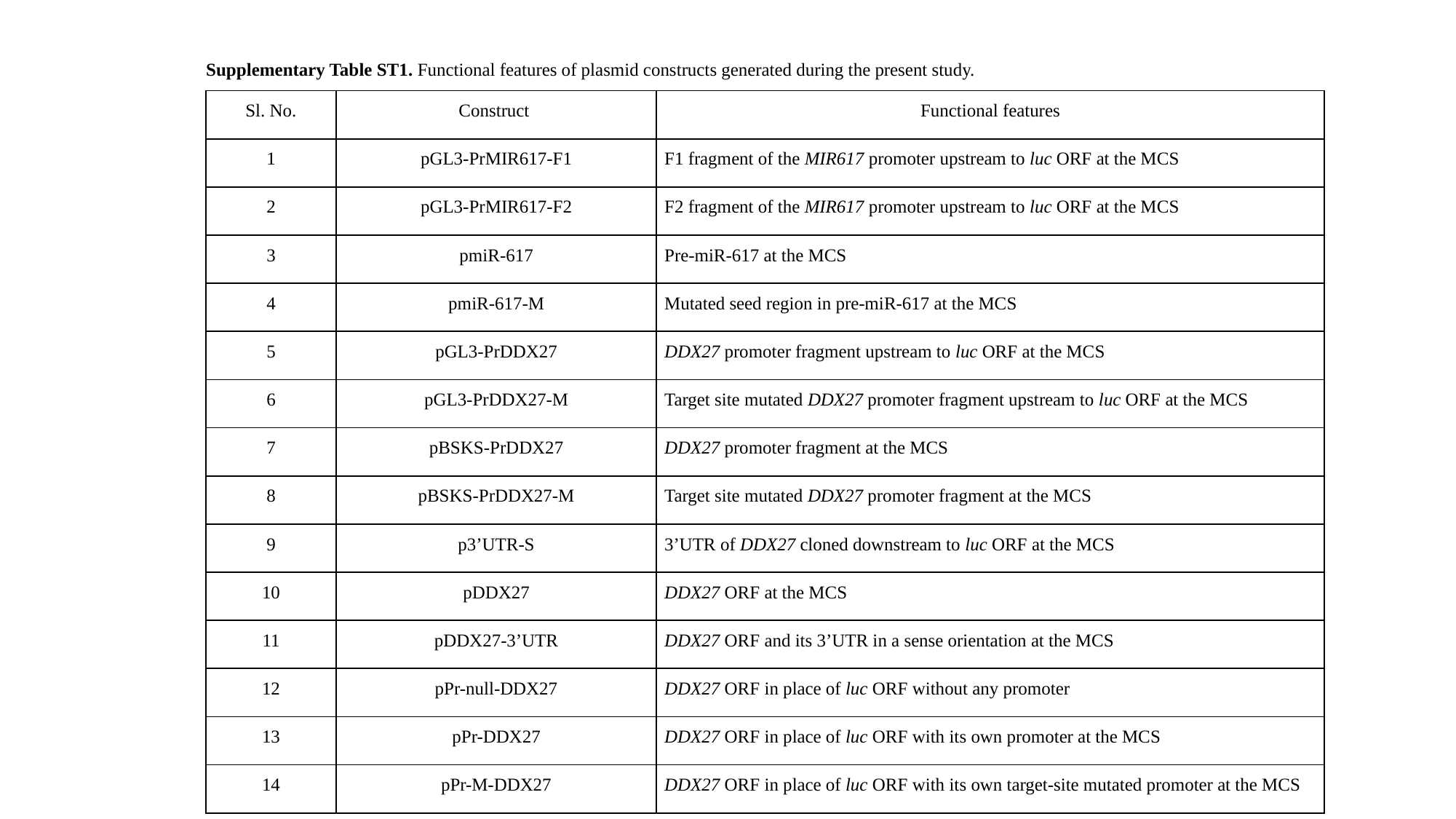

Supplementary Table ST1. Functional features of plasmid constructs generated during the present study.
| Sl. No. | Construct | Functional features |
| --- | --- | --- |
| 1 | pGL3-PrMIR617-F1 | F1 fragment of the MIR617 promoter upstream to luc ORF at the MCS |
| 2 | pGL3-PrMIR617-F2 | F2 fragment of the MIR617 promoter upstream to luc ORF at the MCS |
| 3 | pmiR-617 | Pre-miR-617 at the MCS |
| 4 | pmiR-617-M | Mutated seed region in pre-miR-617 at the MCS |
| 5 | pGL3-PrDDX27 | DDX27 promoter fragment upstream to luc ORF at the MCS |
| 6 | pGL3-PrDDX27-M | Target site mutated DDX27 promoter fragment upstream to luc ORF at the MCS |
| 7 | pBSKS-PrDDX27 | DDX27 promoter fragment at the MCS |
| 8 | pBSKS-PrDDX27-M | Target site mutated DDX27 promoter fragment at the MCS |
| 9 | p3’UTR-S | 3’UTR of DDX27 cloned downstream to luc ORF at the MCS |
| 10 | pDDX27 | DDX27 ORF at the MCS |
| 11 | pDDX27-3’UTR | DDX27 ORF and its 3’UTR in a sense orientation at the MCS |
| 12 | pPr-null-DDX27 | DDX27 ORF in place of luc ORF without any promoter |
| 13 | pPr-DDX27 | DDX27 ORF in place of luc ORF with its own promoter at the MCS |
| 14 | pPr-M-DDX27 | DDX27 ORF in place of luc ORF with its own target-site mutated promoter at the MCS |

## Slide 18
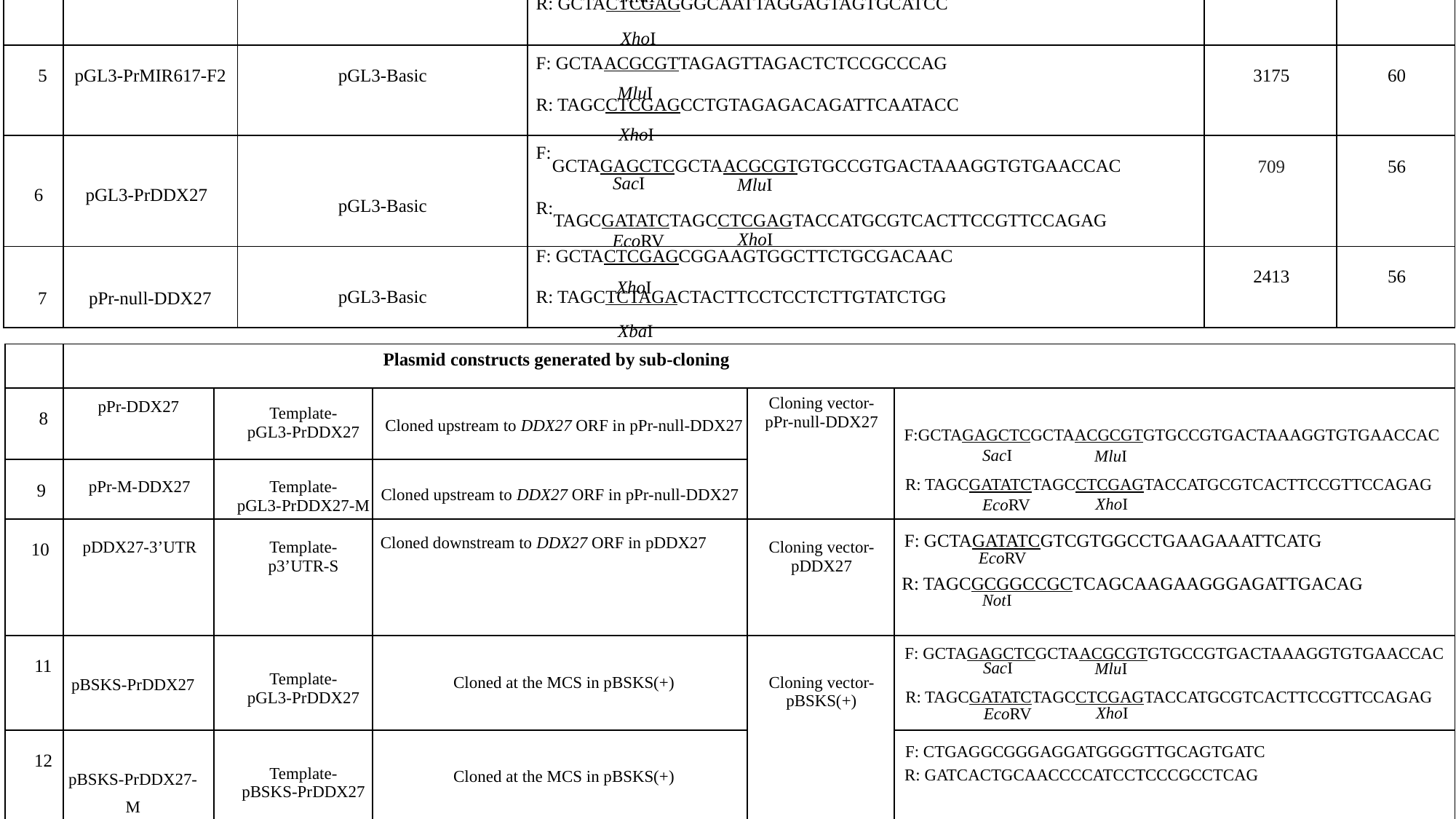

Supplementary Table ST2. Details of the plasmid constructs used in the study.
| Sl. No. | Construct | Cloning vector | Primer sequence (5’ to 3’) | Amplicon size (bp) | Annealing temp. (oC) |
| --- | --- | --- | --- | --- | --- |
| 1 | pmiR-617 | pcDNA3- EGFP | F: ATCGAAGCTTCCTGGAGCCATGTCACGGAAGCTA R: CTAGCTCGAGTGGGATCAACTCTGGTGCTCCTCC | 206 | 56 |
| 2 | pDDX27 | pcDNA3.1(+) | F: GCTAGCGGCCGCGCTAGGTACCCGGAAGTGGCTTCTGCGACAAC R:TAGCGATATCCTACTTCCTCCTCTTGTATCTGG Internal primer 1: CTCCAGTCACCCGCGTGCTGGTG | 2413 | 56 |
| 3 | p3’UTR-S | pMIR- REPORT™ | F: GCTAGAGCTCGTCGTGGCCTGAAGAAATTCATG R: TAGCACGCGTTCAGCAAGAAGGGAGATTGACAG | 186 | 56 |
| 4 | pGL3-PrMIR617-F1 | pGL3-Basic | F: TAGCACGCGTGGGATAATTCTTTCTGGGGGAG R: GCTACTCGAGGGCAATTAGGAGTAGTGCATCC | 825 | 62 |
| 5 | pGL3-PrMIR617-F2 | pGL3-Basic | F: GCTAACGCGTTAGAGTTAGACTCTCCGCCCAG R: TAGCCTCGAGCCTGTAGAGACAGATTCAATACC | 3175 | 60 |
| 6 | pGL3-PrDDX27 | pGL3-Basic | F: R: | 709 | 56 |
| 7 | pPr-null-DDX27 | pGL3-Basic | F: GCTACTCGAGCGGAAGTGGCTTCTGCGACAAC R: TAGCTCTAGACTACTTCCTCCTCTTGTATCTGG | 2413 | 56 |
 HindIII
 XhoI
 KpnI
 EcoRV
 SacI
 MluI
 MluI
 XhoI
 MluI
 XhoI
GCTAGAGCTCGCTAACGCGTGTGCCGTGACTAAAGGTGTGAACCAC
 SacI
 MluI
TAGCGATATCTAGCCTCGAGTACCATGCGTCACTTCCGTTCCAGAG
 XhoI
 EcoRV
 XhoI
 XbaI
| | | | Plasmid constructs generated by sub-cloning | | |
| --- | --- | --- | --- | --- | --- |
| 8 | pPr-DDX27 | Template- pGL3-PrDDX27 | Cloned upstream to DDX27 ORF in pPr-null-DDX27 | Cloning vector- pPr-null-DDX27 | |
| 9 | pPr-M-DDX27 | Template- pGL3-PrDDX27-M | Cloned upstream to DDX27 ORF in pPr-null-DDX27 | | |
| 10 | pDDX27-3’UTR | Template- p3’UTR-S | Cloned downstream to DDX27 ORF in pDDX27 | Cloning vector- pDDX27 | |
| 11 | pBSKS-PrDDX27 | Template- pGL3-PrDDX27 | Cloned at the MCS in pBSKS(+) | Cloning vector- pBSKS(+) | |
| 12 | pBSKS-PrDDX27-M | Template- pBSKS-PrDDX27 | Cloned at the MCS in pBSKS(+) | | |
F:GCTAGAGCTCGCTAACGCGTGTGCCGTGACTAAAGGTGTGAACCAC
 SacI
MluI
R: TAGCGATATCTAGCCTCGAGTACCATGCGTCACTTCCGTTCCAGAG
 XhoI
 EcoRV
 EcoRV
 NotI
F: GCTAGAGCTCGCTAACGCGTGTGCCGTGACTAAAGGTGTGAACCAC
 SacI
 MluI
R: TAGCGATATCTAGCCTCGAGTACCATGCGTCACTTCCGTTCCAGAG
 XhoI
 EcoRV
F: CTGAGGCGGGAGGATGGGGTTGCAGTGATC
R: GATCACTGCAACCCCATCCTCCCGCCTCAG
F: GCTAGATATCGTCGTGGCCTGAAGAAATTCATG
R: TAGCGCGGCCGCTCAGCAAGAAGGGAGATTGACAG
Abbreviations: F, forward primer; R, reverse primer; bp, base pair; and, temp., temperature.

## Slide 19
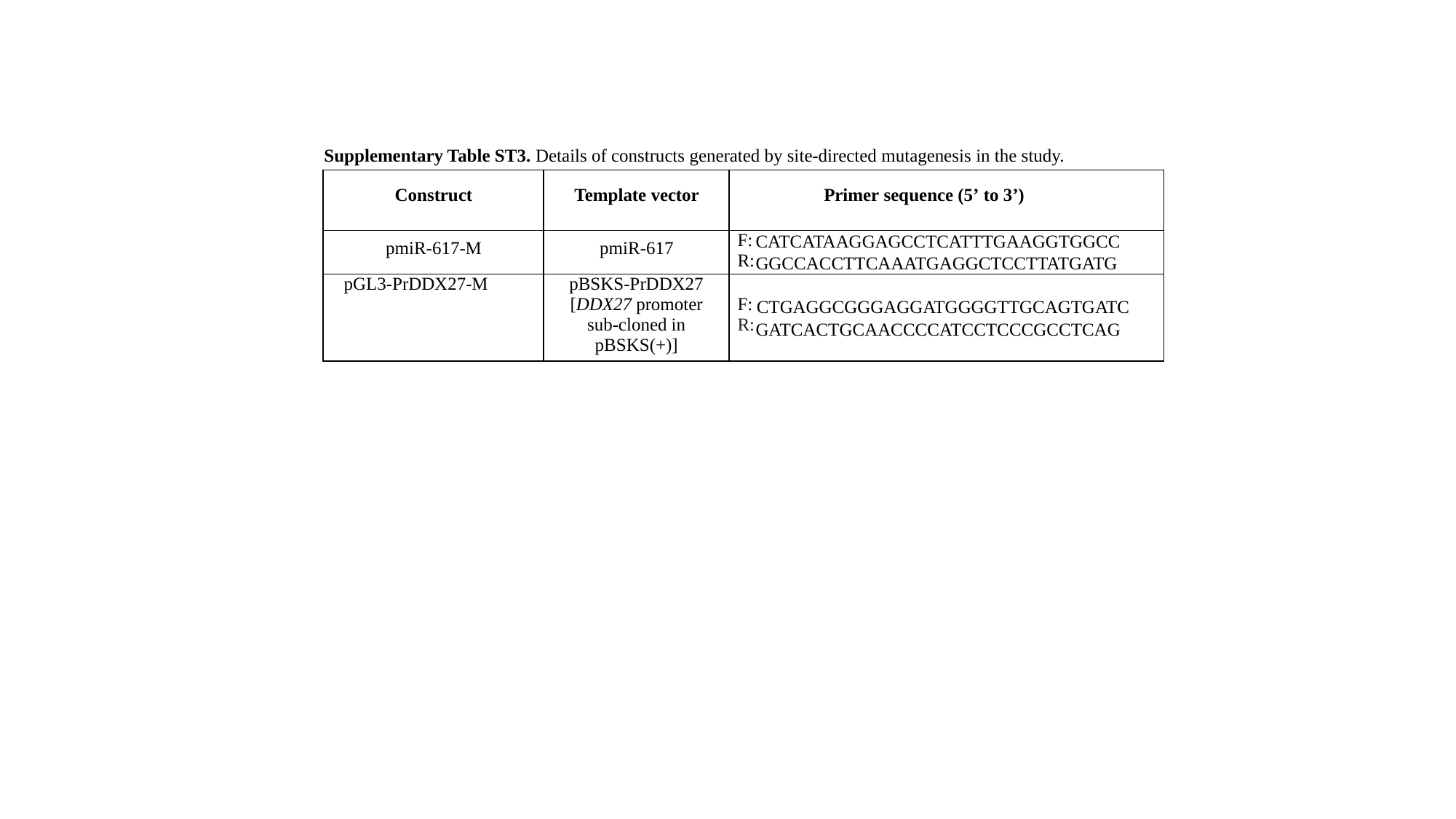

Supplementary Table ST3. Details of constructs generated by site-directed mutagenesis in the study.
| Construct | Template vector | Primer sequence (5’ to 3’) |
| --- | --- | --- |
| pmiR-617-M | pmiR-617 | F: R: |
| pGL3-PrDDX27-M | pBSKS-PrDDX27 [DDX27 promoter sub-cloned in pBSKS(+)] | F: R: |
CATCATAAGGAGCCTCATTTGAAGGTGGCC
GGCCACCTTCAAATGAGGCTCCTTATGATG
CTGAGGCGGGAGGATGGGGTTGCAGTGATC
GATCACTGCAACCCCATCCTCCCGCCTCAG

## Slide 20
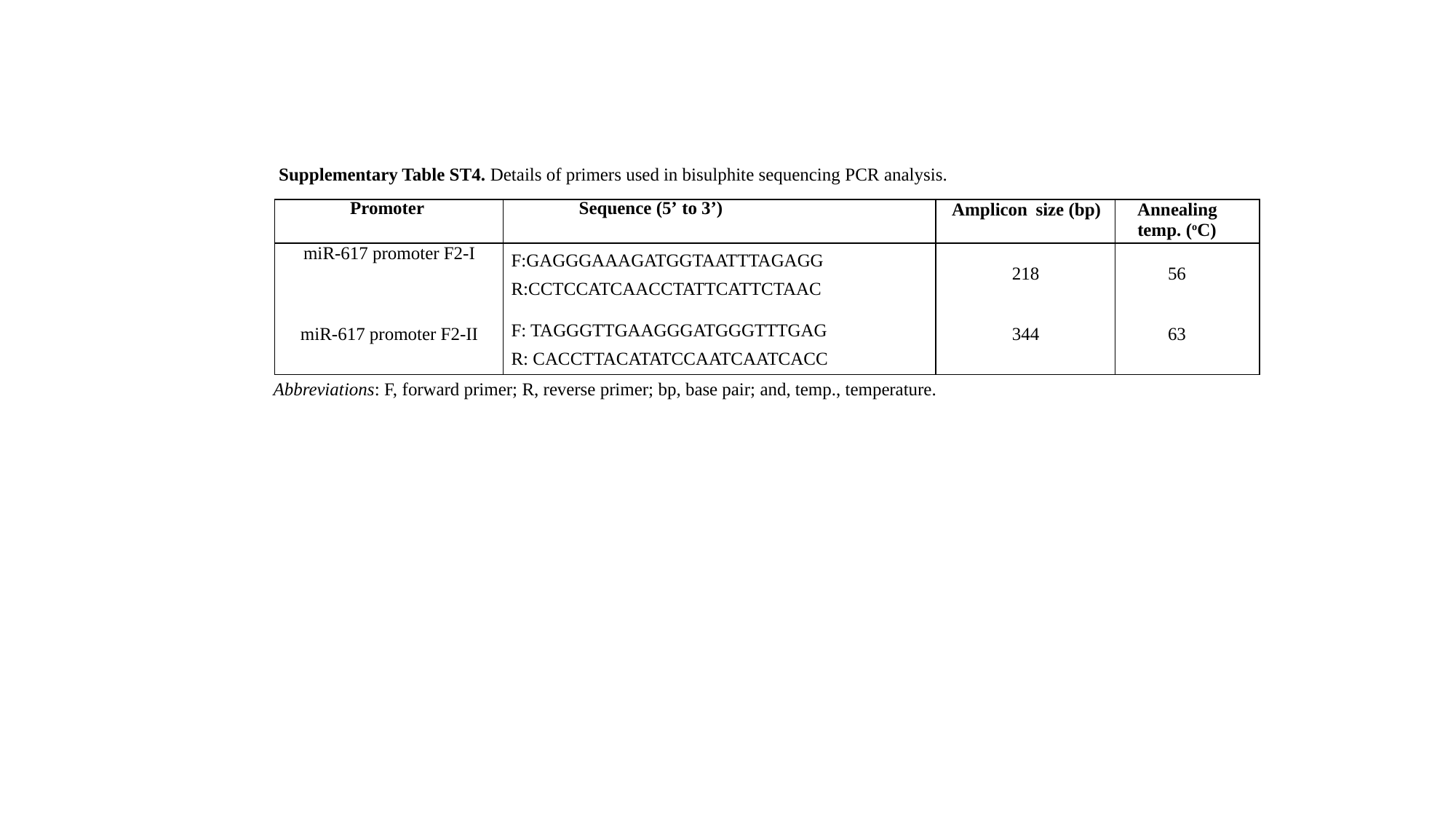

Supplementary Table ST4. Details of primers used in bisulphite sequencing PCR analysis.
| Promoter | Sequence (5’ to 3’) | Amplicon size (bp) | Annealing temp. (oC) |
| --- | --- | --- | --- |
| miR-617 promoter F2-I miR-617 promoter F2-II | F:GAGGGAAAGATGGTAATTTAGAGG R:CCTCCATCAACCTATTCATTCTAAC F: TAGGGTTGAAGGGATGGGTTTGAG R: CACCTTACATATCCAATCAATCACC | 218 344 | 56 63 |
Abbreviations: F, forward primer; R, reverse primer; bp, base pair; and, temp., temperature.

## Slide 21
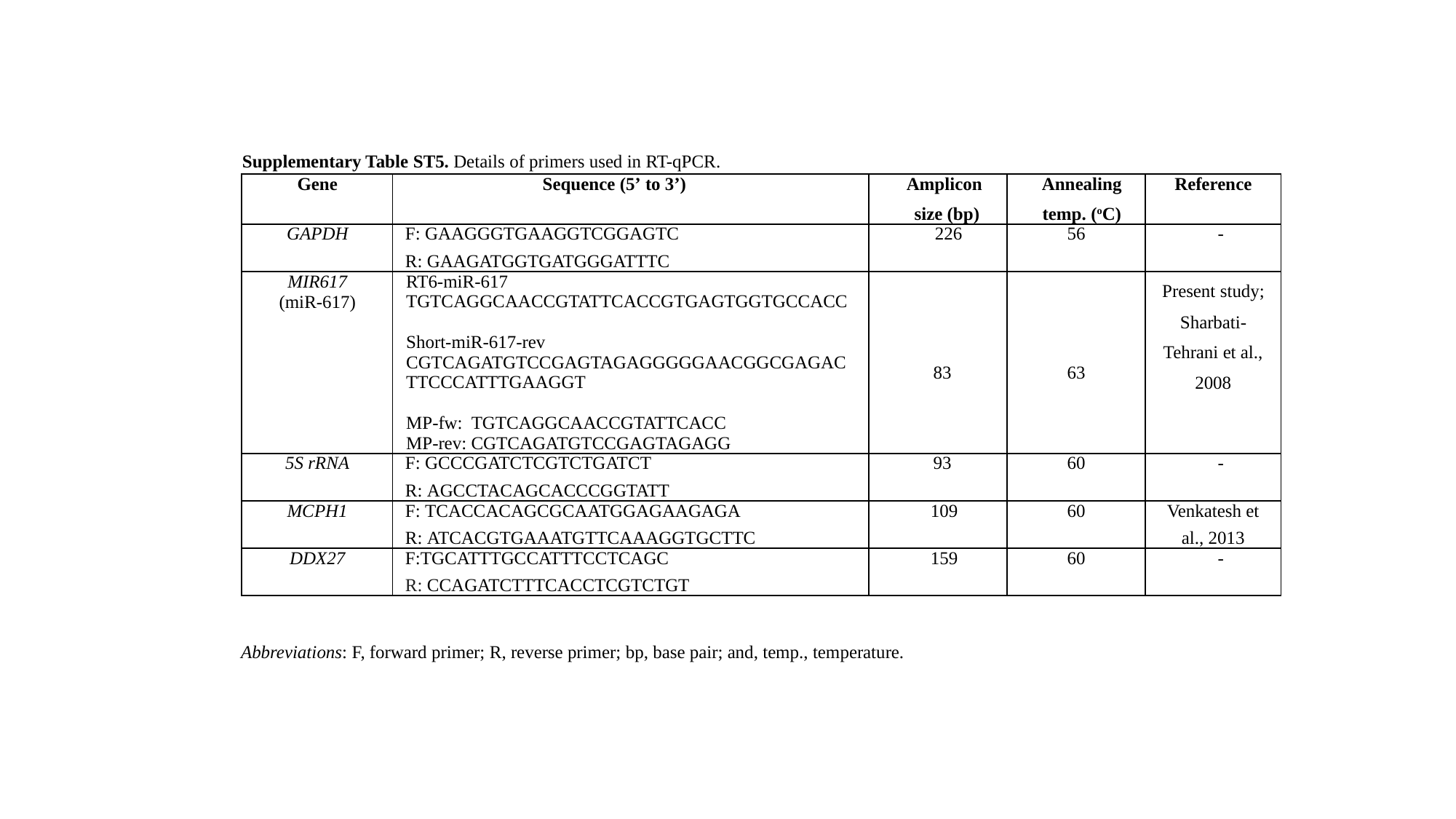

Supplementary Table ST5. Details of primers used in RT-qPCR.
| Gene | Sequence (5’ to 3’) | Amplicon size (bp) | Annealing temp. (oC) | Reference |
| --- | --- | --- | --- | --- |
| GAPDH | F: GAAGGGTGAAGGTCGGAGTC R: GAAGATGGTGATGGGATTTC | 226 | 56 | - |
| MIR617 (miR-617) | RT6-miR-617 TGTCAGGCAACCGTATTCACCGTGAGTGGTGCCACC Short-miR-617-rev CGTCAGATGTCCGAGTAGAGGGGGAACGGCGAGACTTCCCATTTGAAGGT MP-fw: TGTCAGGCAACCGTATTCACC MP-rev: CGTCAGATGTCCGAGTAGAGG | 83 | 63 | Present study; Sharbati- Tehrani et al., 2008 |
| 5S rRNA | F: GCCCGATCTCGTCTGATCT R: AGCCTACAGCACCCGGTATT | 93 | 60 | - |
| MCPH1 | F: TCACCACAGCGCAATGGAGAAGAGA R: ATCACGTGAAATGTTCAAAGGTGCTTC | 109 | 60 | Venkatesh et al., 2013 |
| DDX27 | F:TGCATTTGCCATTTCCTCAGC R: CCAGATCTTTCACCTCGTCTGT | 159 | 60 | - |
Abbreviations: F, forward primer; R, reverse primer; bp, base pair; and, temp., temperature.

## Slide 22
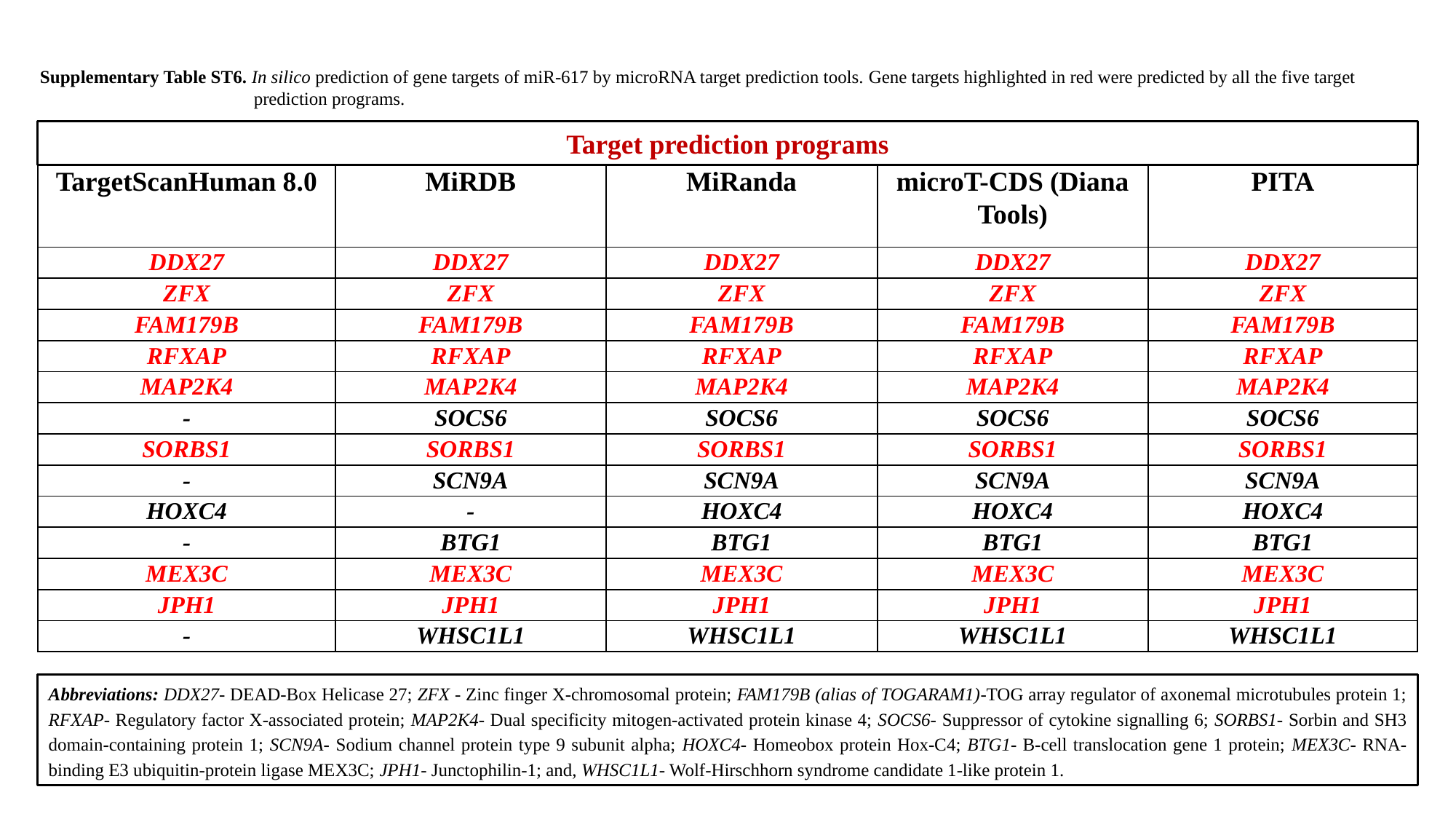

Supplementary Table ST6. In silico prediction of gene targets of miR-617 by microRNA target prediction tools. Gene targets highlighted in red were predicted by all the five target
 prediction programs.
Target prediction programs
| TargetScanHuman 8.0 | MiRDB | MiRanda | microT-CDS (Diana Tools) | PITA |
| --- | --- | --- | --- | --- |
| DDX27 | DDX27 | DDX27 | DDX27 | DDX27 |
| ZFX | ZFX | ZFX | ZFX | ZFX |
| FAM179B | FAM179B | FAM179B | FAM179B | FAM179B |
| RFXAP | RFXAP | RFXAP | RFXAP | RFXAP |
| MAP2K4 | MAP2K4 | MAP2K4 | MAP2K4 | MAP2K4 |
| - | SOCS6 | SOCS6 | SOCS6 | SOCS6 |
| SORBS1 | SORBS1 | SORBS1 | SORBS1 | SORBS1 |
| - | SCN9A | SCN9A | SCN9A | SCN9A |
| HOXC4 | - | HOXC4 | HOXC4 | HOXC4 |
| - | BTG1 | BTG1 | BTG1 | BTG1 |
| MEX3C | MEX3C | MEX3C | MEX3C | MEX3C |
| JPH1 | JPH1 | JPH1 | JPH1 | JPH1 |
| - | WHSC1L1 | WHSC1L1 | WHSC1L1 | WHSC1L1 |
Abbreviations: DDX27- DEAD-Box Helicase 27; ZFX - Zinc finger X-chromosomal protein; FAM179B (alias of TOGARAM1)-TOG array regulator of axonemal microtubules protein 1; RFXAP- Regulatory factor X-associated protein; MAP2K4- Dual specificity mitogen-activated protein kinase 4; SOCS6- Suppressor of cytokine signalling 6; SORBS1- Sorbin and SH3 domain-containing protein 1; SCN9A- Sodium channel protein type 9 subunit alpha; HOXC4- Homeobox protein Hox-C4; BTG1- B-cell translocation gene 1 protein; MEX3C- RNA-binding E3 ubiquitin-protein ligase MEX3C; JPH1- Junctophilin-1; and, WHSC1L1- Wolf-Hirschhorn syndrome candidate 1-like protein 1.

## Slide 23
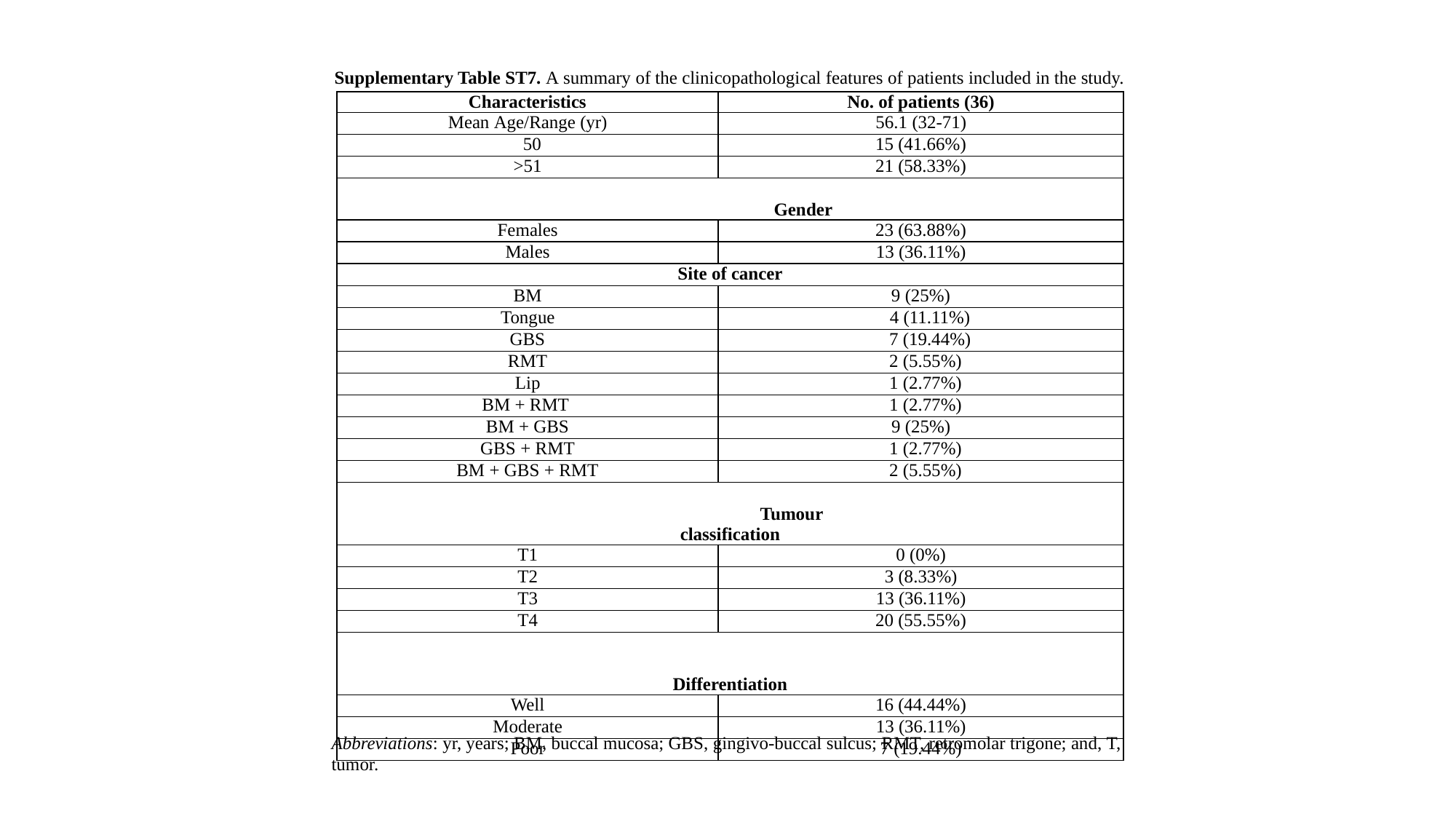

Supplementary Table ST7. A summary of the clinicopathological features of patients included in the study.
| Characteristics | No. of patients (36) |
| --- | --- |
| Mean Age/Range (yr) | 56.1 (32-71) |
| 50 | 15 (41.66%) |
| >51 | 21 (58.33%) |
| Gender | |
| Females | 23 (63.88%) |
| Males | 13 (36.11%) |
| Site of cancer | |
| BM | 9 (25%) |
| Tongue | 4 (11.11%) |
| GBS | 7 (19.44%) |
| RMT | 2 (5.55%) |
| Lip | 1 (2.77%) |
| BM + RMT | 1 (2.77%) |
| BM + GBS | 9 (25%) |
| GBS + RMT | 1 (2.77%) |
| BM + GBS + RMT | 2 (5.55%) |
| Tumour classification | |
| T1 | 0 (0%) |
| T2 | 3 (8.33%) |
| T3 | 13 (36.11%) |
| T4 | 20 (55.55%) |
| Differentiation | |
| Well | 16 (44.44%) |
| Moderate | 13 (36.11%) |
| Poor | 7 (19.44%) |
Abbreviations: yr, years; BM, buccal mucosa; GBS, gingivo-buccal sulcus; RMT, retromolar trigone; and, T, tumor.

## Slide 24
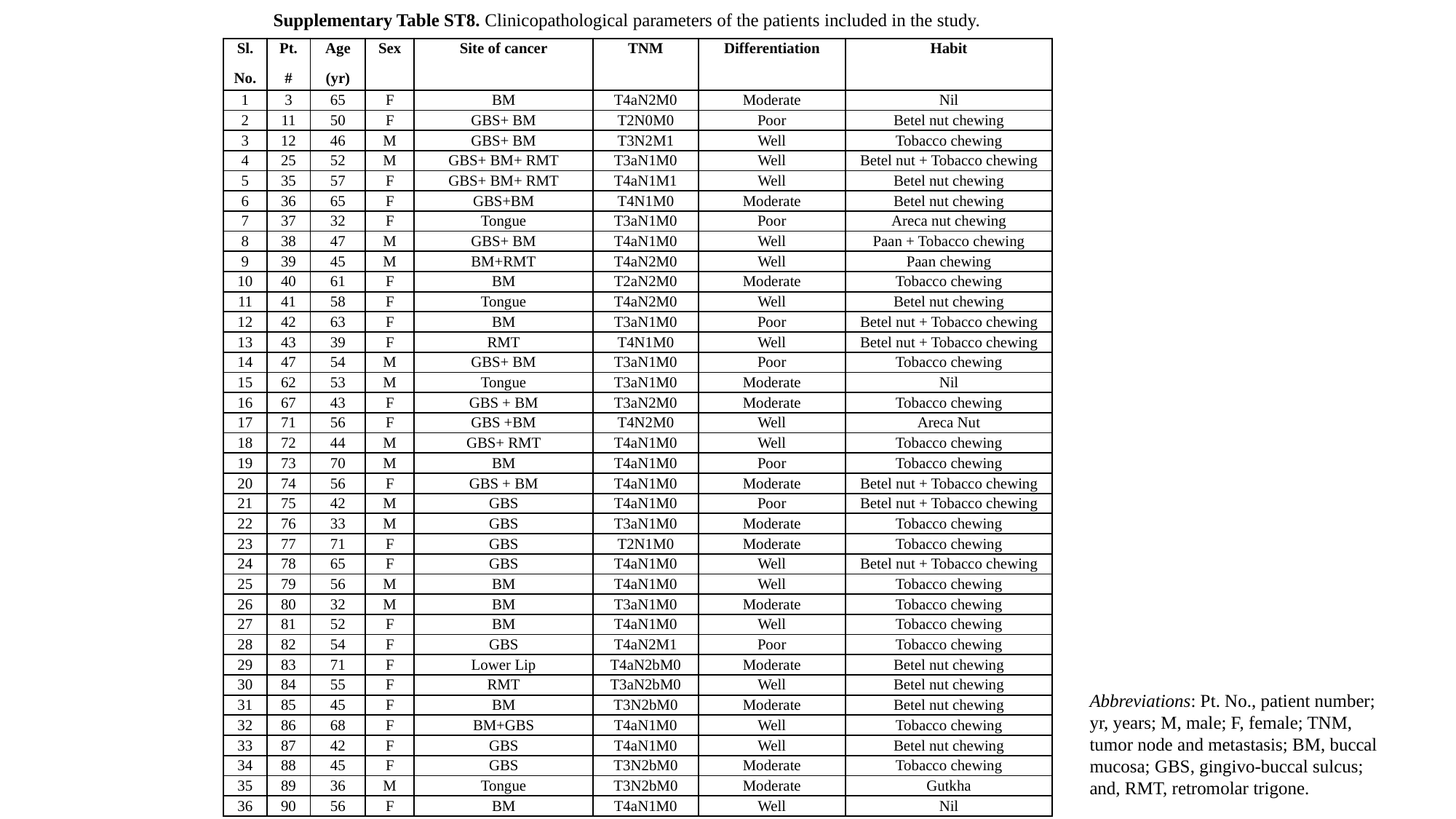

Supplementary Table ST8. Clinicopathological parameters of the patients included in the study.
| Sl. No. | Pt. # | Age (yr) | Sex | Site of cancer | TNM | Differentiation | Habit |
| --- | --- | --- | --- | --- | --- | --- | --- |
| 1 | 3 | 65 | F | BM | T4aN2M0 | Moderate | Nil |
| 2 | 11 | 50 | F | GBS+ BM | T2N0M0 | Poor | Betel nut chewing |
| 3 | 12 | 46 | M | GBS+ BM | T3N2M1 | Well | Tobacco chewing |
| 4 | 25 | 52 | M | GBS+ BM+ RMT | T3aN1M0 | Well | Betel nut + Tobacco chewing |
| 5 | 35 | 57 | F | GBS+ BM+ RMT | T4aN1M1 | Well | Betel nut chewing |
| 6 | 36 | 65 | F | GBS+BM | T4N1M0 | Moderate | Betel nut chewing |
| 7 | 37 | 32 | F | Tongue | T3aN1M0 | Poor | Areca nut chewing |
| 8 | 38 | 47 | M | GBS+ BM | T4aN1M0 | Well | Paan + Tobacco chewing |
| 9 | 39 | 45 | M | BM+RMT | T4aN2M0 | Well | Paan chewing |
| 10 | 40 | 61 | F | BM | T2aN2M0 | Moderate | Tobacco chewing |
| 11 | 41 | 58 | F | Tongue | T4aN2M0 | Well | Betel nut chewing |
| 12 | 42 | 63 | F | BM | T3aN1M0 | Poor | Betel nut + Tobacco chewing |
| 13 | 43 | 39 | F | RMT | T4N1M0 | Well | Betel nut + Tobacco chewing |
| 14 | 47 | 54 | M | GBS+ BM | T3aN1M0 | Poor | Tobacco chewing |
| 15 | 62 | 53 | M | Tongue | T3aN1M0 | Moderate | Nil |
| 16 | 67 | 43 | F | GBS + BM | T3aN2M0 | Moderate | Tobacco chewing |
| 17 | 71 | 56 | F | GBS +BM | T4N2M0 | Well | Areca Nut |
| 18 | 72 | 44 | M | GBS+ RMT | T4aN1M0 | Well | Tobacco chewing |
| 19 | 73 | 70 | M | BM | T4aN1M0 | Poor | Tobacco chewing |
| 20 | 74 | 56 | F | GBS + BM | T4aN1M0 | Moderate | Betel nut + Tobacco chewing |
| 21 | 75 | 42 | M | GBS | T4aN1M0 | Poor | Betel nut + Tobacco chewing |
| 22 | 76 | 33 | M | GBS | T3aN1M0 | Moderate | Tobacco chewing |
| 23 | 77 | 71 | F | GBS | T2N1M0 | Moderate | Tobacco chewing |
| 24 | 78 | 65 | F | GBS | T4aN1M0 | Well | Betel nut + Tobacco chewing |
| 25 | 79 | 56 | M | BM | T4aN1M0 | Well | Tobacco chewing |
| 26 | 80 | 32 | M | BM | T3aN1M0 | Moderate | Tobacco chewing |
| 27 | 81 | 52 | F | BM | T4aN1M0 | Well | Tobacco chewing |
| 28 | 82 | 54 | F | GBS | T4aN2M1 | Poor | Tobacco chewing |
| 29 | 83 | 71 | F | Lower Lip | T4aN2bM0 | Moderate | Betel nut chewing |
| 30 | 84 | 55 | F | RMT | T3aN2bM0 | Well | Betel nut chewing |
| 31 | 85 | 45 | F | BM | T3N2bM0 | Moderate | Betel nut chewing |
| 32 | 86 | 68 | F | BM+GBS | T4aN1M0 | Well | Tobacco chewing |
| 33 | 87 | 42 | F | GBS | T4aN1M0 | Well | Betel nut chewing |
| 34 | 88 | 45 | F | GBS | T3N2bM0 | Moderate | Tobacco chewing |
| 35 | 89 | 36 | M | Tongue | T3N2bM0 | Moderate | Gutkha |
| 36 | 90 | 56 | F | BM | T4aN1M0 | Well | Nil |
Abbreviations: Pt. No., patient number; yr, years; M, male; F, female; TNM, tumor node and metastasis; BM, buccal mucosa; GBS, gingivo-buccal sulcus; and, RMT, retromolar trigone.

## Slide 25
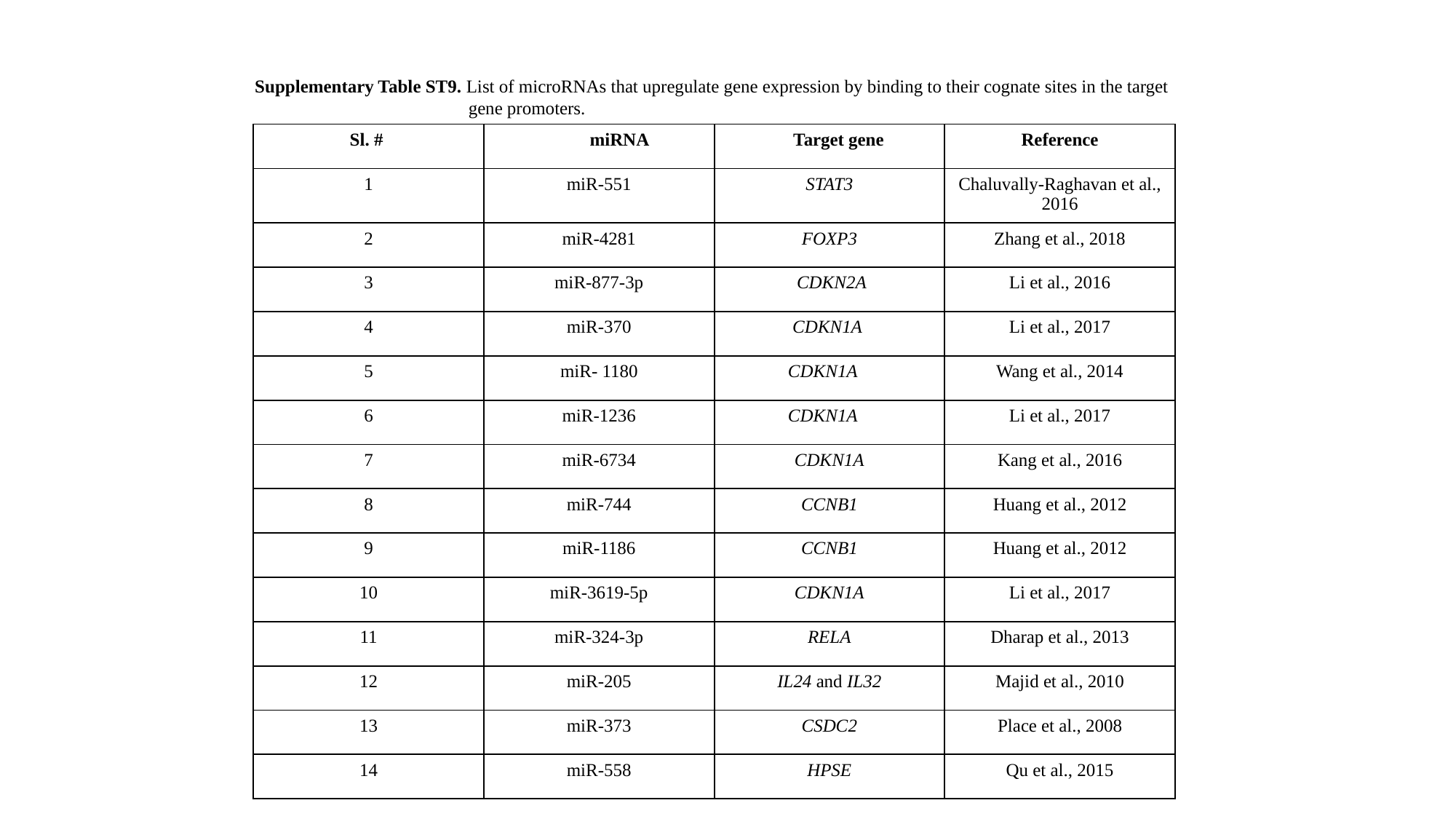

Supplementary Table ST9. List of microRNAs that upregulate gene expression by binding to their cognate sites in the target
 gene promoters.
| Sl. # | miRNA | Target gene | Reference |
| --- | --- | --- | --- |
| 1 | miR-551 | STAT3 | Chaluvally-Raghavan et al., 2016 |
| 2 | miR-4281 | FOXP3 | Zhang et al., 2018 |
| 3 | miR-877-3p | CDKN2A | Li et al., 2016 |
| 4 | miR-370 | CDKN1A | Li et al., 2017 |
| 5 | miR- 1180 | CDKN1A | Wang et al., 2014 |
| 6 | miR-1236 | CDKN1A | Li et al., 2017 |
| 7 | miR-6734 | CDKN1A | Kang et al., 2016 |
| 8 | miR-744 | CCNB1 | Huang et al., 2012 |
| 9 | miR-1186 | CCNB1 | Huang et al., 2012 |
| 10 | miR-3619-5p | CDKN1A | Li et al., 2017 |
| 11 | miR-324-3p | RELA | Dharap et al., 2013 |
| 12 | miR-205 | IL24 and IL32 | Majid et al., 2010 |
| 13 | miR-373 | CSDC2 | Place et al., 2008 |
| 14 | miR-558 | HPSE | Qu et al., 2015 |
